# Emotional experiences of reading health educational manga encouraging behavioral changes: a non-randomized controlled trial

**DOI:** 10.1080/21642850.2021.1921583

**Published:** 2021-04-30

**Authors:** Takashi Shimazaki, Misa Iio, Hiroaki Uechi, Koji Takenaka

**Affiliations:** a Department of Health & Physical Education, Faculty of Humanities, Sophia University, Tokyo, Japan; b College of Nursing, Kanto-Gakuin University, Yokohama, Japan; c Faculty of Education, Yamaguchi University, Yamaguchi, Japan; d Faculty of Human Sciences, Waseda University, Tokyo, Japan

**Keywords:** Emotional experience, educational manga, behavior change, mediator

## Abstract

**Objective:**

Educational health manga are used to promote health behavior change. This study thus seeks to achieve the following objectives: identify the emotional experience of reading educational health manga, understand the effects of facilitating the psychological mediators of behavior change through emotional experiences, and compare the effectiveness of educational health manga with conventional materials.

**Design:**

A non-randomized controlled trial with six conditions was conducted. Target behaviors were physical activity and healthy diet. Individuals aged 20–69 years (*n* = 1,680) were assigned three manga intervention conditions and three control conditions (visual image-based, narrative text, and general text material).

**Main Outcome Measures:**

Participants were asked about their emotional experience while reading either intervention material or control material and its perceived positive influence on enhancing psychological mediators.

**Results:**

Four factors relevant to emotional experiences were identified: risk perception, familiarity, satisfaction, and realism. Emotional experience strongly predicted the psychological mediators of behavior change. Analysis of differences among the six conditions revealed that exposure to educational health manga led to greater satisfaction and increased knowledge. Participants assigned manga conditions experienced superior emotional experiences and outcomes.

**Conclusions:**

Those assigned manga conditions experienced superior emotional experiences and outcomes. The efficacy of educational health manga in encouraging psychological mediators of change was confirmed.

**Trial registration:** UMIN Japan identifier: UMIN000034369.

Research has revealed that physical activity and a healthy diet play important roles in the promotion of physical, mental, and social health (World Health Organization [WHO], 2014). Previous studies have reported that promoting physical activity and healthy diet can aid in the prevention of non-communicable diseases, such as obesity (Cecchini et al., 2010), diabetes (Hu, 2011), cardiovascular diseases (Benjamin et al., 2019), cancer (Kushi et al., 2012), and mental health problems (Lai et al., 2014; Penedo & Dahn, 2005). Such diseases have constituted important health issues in Japan and abroad, including developed and developing countries (WHO, 2017). However, providing health education for high-risk populations that lack self-efficacy (SE) and supporting the development of positive intentions for physical activity and healthy diet are significant challenges (Fletcher, Behrens, & Domina, 2008).

Currently, strategies for promoting health behavior, which can directly inform evidence-based information and health promotion guidelines, are lacking (Brawley & Latimer, 2007). As a method for communicating health-related messages through various media considered appropriate for target audiences, messaging can play a significant role in disseminating messages for health behavior change (Latimer, Brawley, & Bassett, 2010). The concept of entertainment education (Singhal, Wang, & Roger, 2013), which provides educational information to increase psychological mediators for desirable behavior change through entertainment media, such as radio, television, popular music, films, digital games, and comics, is a potential effective communication strategy for delivering health messages to high-risk populations (Literat & Chen, 2014; Reinermann, Lubjuhn, Bouman, & Singhal, 2014; Volk et al., 2008).

Many educational institutions and programs have utilized manga, i.e. when Japanese-style comics are used as an effective learning tool, they are called educational manga. The graphic medicine approach, which indicates the use of manga as a means of providing medical information, is widely used in health care and patient education settings (Green & Myers, 2010). A previous systematic review revealed that the application of manga positively influenced health care settings (Branscum & Sharma, 2009). Several randomized controlled trials reported a small effect on initiatives of health behavior change for promoting general health (Junhasavasdikul et al., 2017) and for preventing back pain (Kovacs et al., 2011). However, other studies challenged the application of health promotion manga or comics for a range of patient education or health education initiatives, such as programs for promoting physical activity and healthy diet (Baranowski et al., 2002; Branscum, Sharma, Wang, Wilson, & Rojas-Guyler, 2013a, 2013b; Leung et al., 2017; Leung, Tripicchio, Agaronov, & Hou, 2014; Manes, Liu, Burke, & Dworkin, 2014); the prevention infectious diseases (Bieri, Yuan et al., 2013; Yuan, Manderson, Tempongko, Wei, & Aiguo, 2000), HIV/AIDS (Dworkin et al., 2013; Gillmore et al., 1997), and cancer (Lyzun & McMullen, 2009; Risi et al., 2004); immunization (Diamond et al., 2016; Muzumdar & Nania, 2015; Muzumdar & Pantaleo, 2017); smoking cessation (Bush et al., 2005); reduction of disease-related stigma (el-Setouhy & Rio, 2003), education on pesticide exposure and health (Liebman, Juárez, Leyva, & Corona, 2007); prevention of accidents among children (Cardenas & Simons-Morton, 1993); and patient education (Maxwell, Simmons, Franklin, Arnold, & Pall, 2014; Shigehatake et al., 2014). In addition, several trials reported that manga is an effective tool for health messaging as a subsidiary material in programs for health promotion (e.g. as part of a toolkit; Alam, Elwyn, Percac-Lima, Grande, & Durand, 2016; Bellingham & Gillies, 1993).

Empirical practice-based measures have revealed that using health educational manga is supported by three theoretical frameworks: social cognitive theory of modeling, narrative or story-telling approach, and effects of visual imagery (Shimazaki, Matsushita, Iio, & Takenaka, 2018). Observational learning about health behaviors practiced by characters in a manga comic may operate via a modeling effect (Bandura, 2001). Narrative or story-telling-based text may increase acceptability and readability in implementing health messaging (Dahlstrom, 2014; Kreuter et al., 2007). Previous studies have established the effects of visual imagery that employs cartoons and infographics to increase sensory understanding (Houts, Doak, Doak, & Loscalzo, 2006; Scott, Fawkner, Oliver, & Murray, 2016). The use of visual imagery may enhance an understanding of difficult and complex medical information (Gebarski et al., 2013; Kassai et al., 2016; Moll, Wright, Jeffrey, Goode, & Humberstone, 1977; Tae et al., 2012). Furthermore, some research suggests that decoding health messages via certain entertainment mediums, such as manga, elicits emotional experiences that contribute to behavior change (Sood, Riley, & Alarcon, 2017).

It is well-established that emotional positive affect facilitates physical activity and a healthy diet (Van Cappellen, Rice, Catalino, & Fredrickson, 2018). A comprehensive review of research shows that affective states resulting from exposure to health messages are essential to decision-making and behavior change (Ferrer & Mendes, 2018). When receiving health messages, participants might feel several emotions (fear, sadness, and happiness) and moods (negative and positive affects) simultaneously (Lerner, Li, Valdesolo, & Kassam, 2015). Previous studies have hypothesized that readers undergo various emotional experiences, including fun, enjoyment, expected outcomes of healthy and unhealthy lifestyles, risk perception, anxiety related to an unhealthy lifestyle, familiarity, and emotional transportation, so as to understand characters’ feelings, emotional involvement with the story, feelings of suspense, identification with characters’ feelings, and the satisfaction of reading (Sood et al., 2017). Extensive endeavors have examined the use of manga positively influencing behavior change in health care settings. However, relatively little is known about the underlying psychological mechanisms of health educational manga to facilitate healthy lifestyle behavior changes. Furthermore, no previous studies have examined the differences in the effectiveness of increasing mediator variables for engagement in physical activity and healthy diet behavior changes among approaches employing manga, visual image-based materials, narrative story-telling text, and general health information.

This study hypothesized that a combination of three different effects are involved in inducing appropriate emotional experiences while participants read health educational manga. Emotional experiences may facilitate SE and behavioral intentions of desirable behavior change. Furthermore, previous research has also demonstrated that employing educational manga in health promotion settings may enhance psychological mediators to engage in health behavior changes (Sood et al., 2017).

The current study intended (1) to identify emotional experiences related to reading physical activity and healthy diet focused educational manga; (2) to confirm the effects of emotional experiences on facilitating the psychological mediators of physical activity and healthy diet changes; and (3) to examine the differences in the effectiveness of educational manga, visual image-based material, narrative story-telling material, and general information.

## Methods

### Trial design and participants

An Internet based non-randomized controlled trial with post-assessment only in a physical activity and healthy diet educational manga (PAHDEM) was conducted in Japan. The results were reported using the CONSORT 2010 frameworks (see Additional file 1). The trial was conducted on the online platform of Cross Marketing Inc., a Japanese online research provider. Eligibility criteria were used to select participants aged 20–69 years who agreed to participate in the study after reading a descriptive informed consent form and agreeing to read the intervention materials; manipulation-checking questions were utilized to confirm the participants had complied with the latter. Participants providing informed consent were presented with the intervention materials. Further, they were required to answer a manipulation-checking question. They had to indicate how much of the given health information they had read by using a 5-point Likert scale (1 = none at all, 5 = read a lot). A total of 62 participants who indicated they had read ‘none at all’ were excluded from the first recruitment. Consequently, additional recruitment was conducted to recruit more participants, who were given online coupons from the online research provider as compensation.

**Figure 1.**
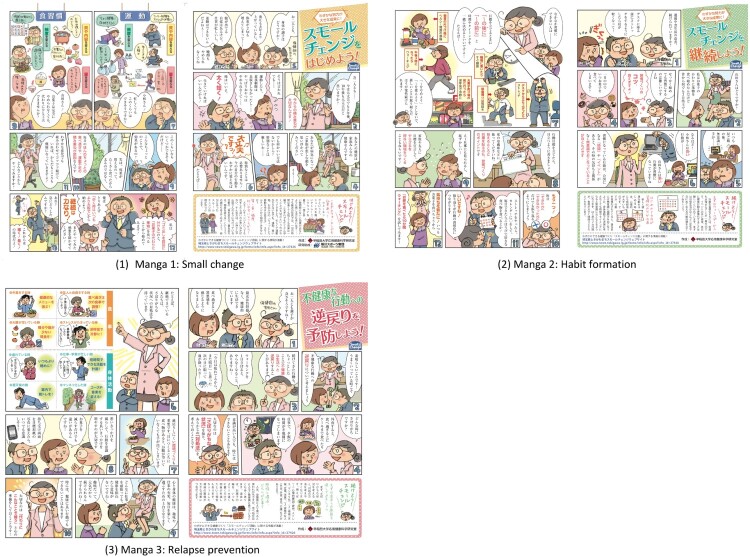
Physical activity and healthy diet educational manga. Note. English translations of dialog with original Japanese version are provided in Additional file 2.

Age- and gender-related biases in the intervention effect related to stigmas associated with using educational manga and the possibility of improvement in physical activity and health diet were considered in assigning participants to the intervention or control condition. Branscum and Sharma (2009) suggested that many older people felt that manga was not a serious medium for educational materials. Moreover, Hiller, Schatz, and Drexler (2017) noted that women were more likely to be health conscious. In addition, men display more potential to improve inactivity than women do. Therefore, age and gender were adjusted to avoid bias across the six conditions. Allocation was conducted by Cross Marketing Inc. The participants were assigned to six different conditions including three intervention conditions and three control conditions. The participants were blinded to their assignment to either the intervention or control groups until they had completed the questionnaire. The three intervention groups were provided with three types of manga as follows. Manga 1 pertains to the adaption of a new healthy behavior according to the theory of small change (Hill, Peters, & Wyatt, 2009; Lutes & Steinbaugh, 2010). Manga 2 discusses habit formation (Gardner, Lally, & Wardle, 2012), and Manga 3 focuses on relapse prevention (Marlatt & Donovan, 2005) of physical inactivity and unhealthy eating styles. The intervention materials are presented in Figure 1. The full English text can be found in Additional file 2. Moreover, three control conditions were included: Control 1: visual image information that conveyed tips regarding physical activity and healthy diet in daily living; Control 2: Text-based narrative information that was used only in the dialog of Manga; and Control 3: General text material that explained the importance of small lifestyle changes in daily living. The control stimuli are also displayed in Additional file 2. The participants were presented with assigned stimuli on their computer screen. After reading the intervention materials, they advanced to the questionnaire page. To prevent participants answering the questionnaire before reading the material, the questionnaire website could only be accessed after the intervention material had been displayed for one minute. After completing the questionnaire, the manga materials were displayed.

To the best of the authors’ knowledge, no previous systematic reviews that employed meta-analyses have examined studies of the effectiveness of health promotion manga. Previously published randomized control trials of manga in health care settings (Junhasavasdikul et al., 2017; Kovacs et al., 2011) revealed small to medium intervention effects, and thus, this study assumed the same. The sample size was calculated by examining the difference in intervention effects among the six conditions by employing an analysis of covariance (ANCOVA). Power analysis was conducted with the following parameters: number of groups = 6; effect size *f* = 0.1 (i.e. small effect), level of significance = 0.05; and statistical power = 0.9. The results indicated that a minimum of 1,656 participants, i.e. 276 per group was required. This study was conducted by employing an online survey, which was administered by a research provider, and a single assessment method. The risk of dropout was assumed to be low. Consequently, a sample size of 280 participants per group (i.e. a total of 1,680 participants were included (intervention group = 840; control group = 840) was employed.

### Intervention materials

The three manga intervention materials contained three consistent characters: two clients, and a public health nurse. The public health nurse, explained how to start healthy behavior change (Manga 1), provided tips about the duration and habit formation of each activity (Manga 2), and explained how to prevent relapsing to unhealthy lifestyle habits (Manga 3). All three manga materials included two short topics with illustrations of starting behavior change.

Manga 1: small change was used in a previously published case study (Shimazaki et al., 2018). It was conducted in accordance with the small change model (Hill et al., 2009; Lutes & Steinbaugh, 2010), which promotes the low psychological burden and high feasibility of applying healthy behavior in daily life. The effects of brisk walking to enhance cardiovascular health (Loprinzi & Cardinal, 2013) and how to develop an appropriate action plan (Lutes et al., 2012) were discussed. Manga 2: habit formation illustrated how people engage in physical activity and healthy diet continuously. The theory of habit formation was mainly applied to the content (Gardner et al., 2012). An if–then rule related to goal-setting of timing or events was described and the importance of self-monitoring was introduced (Lally & Gardner, 2013; Michie, Abraham, Whittington, McAteer, & Gupta, 2009). Tips about forming habitual physical activity and healthy diet by setting achievable goals on weekdays were provided. Weekdays were emphasized because lifestyle factors are relatively stable during the week. Furthermore, cognitive effort may be gradually reduced after approximately two months of engaging in a new behavior (Lally, van Jaarsveld, Potts, & Wardle, 2010). In accordance with the well-established relapse prevention theory, Manga 3: relapse prevention explained how people relapse from newly formed health habits and relapse to unhealthy lifestyle habits (Marlatt & Donovan, 2005). The topics were consistent in relation to identifying individual and environmental cues related to not adhering to physical activity (Stetson et al., 2005) as well as how to improve the environment to reduce snacking and/or the consumption of junk food (Painter, Wansink, & Hieggelke, 2002).

All three types of manga were considered in the Japanese cultural context according to previous studies, which reported the practices of healthy Japanese individuals toward healthy lifestyles in daily living with a particular focus on physical activity and healthy diet (Shimazaki, Iio, Lee, Konuma, & Takenaka, 2016; Shimazaki, Iio, Lee, Suzuki, et al., 2016).

### Outcome measurement

Participants were asked for information about their gender, age, educational background, current physical activity and healthy diet, marital status, parenting, nursing care, employment status, job type, and working time.

Measured questionnaire was illustrated in Additional file 3. Draft items of emotional experiences were identified. Further, this study referenced a range of review articles (Bertrand, O’Reilly, Denison, Anhang, & Sweat, 2006; Bieri, Gray, Raso, Li, & McManus, 2012; Shen & Han, 2014; Sood et al., 2017) and cross-sectional studies (McNicol, 2017; Quintero Johnson, Harrison, & Quick, 2013; van Leeuwen, Renes, & Leeuwis, 2013) that explored the psychological benefits of entertainment education. The first author reviewed the emotional outcomes revealed in these studies. All authors who specialized in health psychology discussed the hypothesized concepts underlying the study. The participants were asked to assess their feelings while reading health educational manga by employing a 5-point Likert scale for 20 questions. Additionally, the acceptability and usability of the presented health information were tested by employing a previously published scale (Shimazaki, Maeba, Iio, Takenaka, & Kikkawa, 2013) to confirm the criterion-related validity of emotional experiences of health educational manga as conceptualized in this study.

SE and behavioral intention were defined as primary outcomes. SE and intention are well-known predictive factors for health behavior and are able to reflect the impact of intervention in the short term (Ajzen, 1991; Bandura, 1977). A questionnaire was administered in accordance with a well-established scale based on the theory of planned behavior (Francis et al., 2004). The participants responded to three items for each domain using a 5-point Likert scale. Single-item questions were employed to confirm the positive effects of the intervention on secondary outcomes. Secondary outcomes were selected from a previously published study of the outcomes of health educational manga as well as systematic reviews of entertainment education. The participants assessed the mediator variables of changes related to healthy behavior, such as motivation, attitude, subjective norm, knowledge, and recall via a self-assessment questionnaire with five items rated using a five-point Likert-type scale. No changes in research procedure and outcome measurement were noted after the commencement of the PAHDEM trial.

### Ethics approval and consent to participate

The present study was approved by Sophia University Regulations for Ethics Committee (reference number: 2018–63). All study procedures were conducted by following the Sophia University Guidelines for ‘Research on human subjects’ and the ethical principles for medical research involving human subjects specified in the Declaration of Helsinki.

### Statistical analysis

Before the main statistical analysis was performed, bias risk was estimated. Chi-squared analysis and effect size (*w*) were calculated to examine the difference in dropout ratio as well as the risk of bias from the demographic characteristics among the six intervention and control conditions. Cohen’s *w* was calculated to assess the magnitude of the bias risk. Small, medium, and large risk corresponded to values of 0.10, 0.30, and 0.50, respectively (Cohen, 1992).

In relation to the authors’ first research aim, the emotional experiences of reading health-related manga material were identified by utilizing the data from the participants assigned to the three intervention conditions (*n* = 840). After confirming the ceiling and floor effects, this study conducted exploratory factor analysis by employing the maximum likelihood method with promax-rotation. The cutoff criteria of factor loading were defined as < 0.40. Reliability was tested using Cronbach’s alpha coefficient and the Spearman–Brown split-half reliability coefficient. Validity was tested by measuring criterion-related validity using Pearson’s correlation analysis with an acceptability–usability scale for health promotion materials. The relationship between emotional experience and psychological mediators of behavior change, i.e. primary and secondary outcomes was examined using structural equation modeling (SEM) to examine the authors’ secondary research aim. The criteria of the model fit index were defined as a goodness of fit index (GFI) value > 0.90, adjusted GFI (AGFI) value > 0.90, comparative fit index (CFI) value > 0.90, root mean square error of approximation (RMSEA) < 1.00, and *R*^2^ of dependent variable of > 0.20 (Hooper, Coughlan, & Mullen, 2008).

In relation to the authors’ third research aim, the differences in the intervention effects among the six intervention and control conditions were examined using ANCOVA. In summary, the demographic characteristics include gender, age, educational background, current physical activity and healthy diet, marital status, parenting, nursing care, employment status, job type, and working time, except when these independent variables were treated as covariates. Furthermore, the manga conditions were adjusted in terms of whether the manga material was readiness-matched or not. When a main effect was observed, Bonferroni’s post-hoc analysis was conducted. Cohen’s *f* was also calculated to identify an intervention effect on changing the psychological mediators of behavior change. Cohen’s *f* was defined as small, medium, and large, corresponding to *f* = 0.10, 0.25, and 0.40, respectively (Cohen, 1992).

IBM SPSS version 24 and Amos version 24 were employed to conduct the statistical analysis. Effect size was calculated R version 3.4.3 with the ‘pwr version 1.2–1’ package to provide the basic function of the power analysis.

## Results

### Demographic characteristics

**Figure 2.**
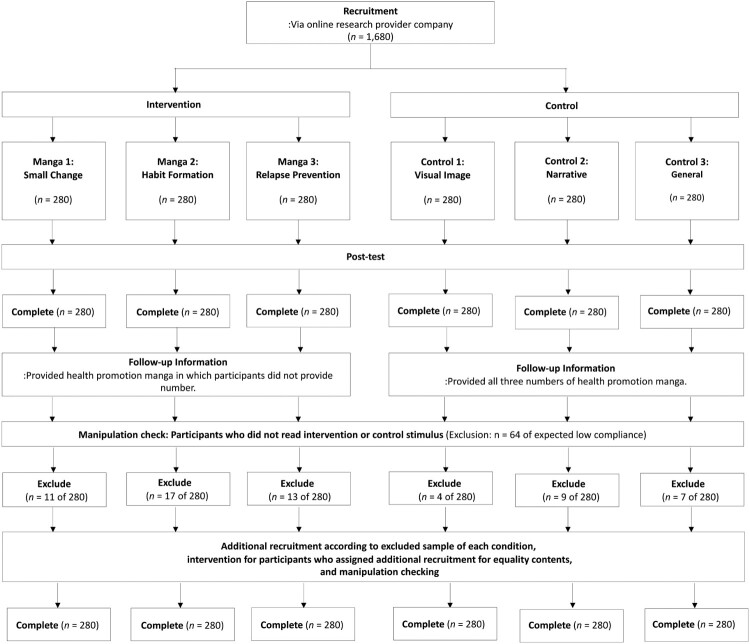
Overview of the present study.

An overview of the study is depicted in Figure 2. Recruitment occurred between 3 October and 28 October 2018 to reach the targeted sample size. The differences in the dropout (i.e. participants who did not read the intervention or control materials) ratio among the six conditions were statistically non-significant with small effect sizes (*χ*^2^ = 9.93, *df* = 5, *w* = 0.08). In Table 1, the participants’ demographic characteristics are presented. There were no significant differences in the demographic characteristics of the participants in the six conditions. The effect sizes were small (*w* = 0.00–0.14). In summary, data of 840 participants each for the intervention and control groups were used for analysis. The participants reported no significant harms and unintended effects.

**Table 1.** Participants’ characteristics.

	Intervention (Manga condition)						Control						*χ*^2^	*df*	*w*
	Small change		Habit formation		Relapse prevention		Visual image		Narrative		General				
	*n*	(%)	*n*	(%)	*n*	(%)	*n*	(%)	*n*	(%)	*n*	(%)			
*Gender*															
Male	140	(50)	140	(50)	140	(50)	140	(50)	140	(50)	140	(50)	0.00	5	0.00
Female	140	(50)	140	(50)	140	(50)	140	(50)	140	(50)	140	(50)			
*Age*															
20–29	56	(20)	56	(20)	56	(20)	56	(20)	56	(20)	56	(20)	0.00	20	0.00
30–39	56	(20)	56	(20)	56	(20)	56	(20)	56	(20)	56	(20)			
40–49	56	(20)	56	(20)	56	(20)	56	(20)	56	(20)	56	(20)			
50–59	56	(20)	56	(20)	56	(20)	56	(20)	56	(20)	56	(20)			
60–69	56	(20)	56	(20)	56	(20)	56	(20)	56	(20)	56	(20)			
*Education (graduation)*															
Junior high or high school	70	(25)	84	(30)	89	(32)	94	(34)	79	(28)	84	(30)	8.78	10	0.07
University	171	(61)	154	(55)	146	(52)	152	(54)	160	(57)	161	(58)			
Graduate school	39	(14)	42	(15)	45	(16)	34	(12)	41	(15)	35	(13)			
*Current physical activity and healthy diet*															
Do not practice	72	(26)	80	(29)	92	(33)	87	(31)	69	(25)	94	(34)	11.63	10	0.08
Practicing but not habitual	140	(50)	135	(48)	123	(44)	125	(45)	150	(54)	126	(45)			
Practicing habitually	68	(24)	65	(23)	65	(23)	68	(24)	61	(22)	60	(21)			
*Marital status*															
Yes	115	(41)	125	(45)	129	(46)	118	(42)	125	(45)	107	(38)	4.80	5	0.05
No	165	(59)	155	(55)	151	(54)	162	(58)	155	(55)	173	(62)			
*Parenting*															
Yes	139	(50)	146	(52)	130	(46)	147	(53)	139	(50)	151	(54)	4.06	5	0.05
No	141	(50)	134	(48)	150	(54)	133	(48)	141	(50)	129	(46)			
*Nursing care*															
No	194	(69)	194	(69)	210	(75)	199	(71)	187	(67)	203	(73)	7.22	10	0.14
Home nursing care	16	(6)	20	(7)	13	(5)	16	(6)	19	(7)	12	(4)			
Live apart nursing care	70	(25)	66	(24)	57	(20)	65	(23)	74	(26)	65	(23)			
*Employment status*															
Permanent employment	119	(43)	134	(48)	103	(37)	122	(44)	117	(42)	116	(41)	32.32	30	0.14
Non-regular employment	57	(20)	41	(15)	60	(21)	57	(20)	66	(24)	51	(18)			
Self-employment	14	(5)	13	(5)	23	(8)	24	(9)	18	(6)	16	(6)			
Housekeeper	51	(18)	44	(16)	43	(15)	44	(16)	39	(14)	60	(21)			
Retirement	12	(4)	13	(5)	15	(5)	9	(3)	12	(4)	10	(4)			
Not working	15	(5)	27	(10)	24	(9)	16	(6)	18	(6)	17	(6)			
No response	12	(4)	8	(3)	12	(4)	8	(3)	10	(4)	10	(4)			
*Working hours per day (hour/day)*															
Legal working hours (<8)	119	(43)	118	(42)	113	(40)	118	(42)	131	(47)	120	(43)	16.06	20	0.10
Acceptable over time working (<12)	60	(21)	61	(22)	68	(24)	75	(27)	60	(21)	58	(21)			
Critical overwork (>12)	9	(3)	9	(3)	4	(1)	10	(4)	10	(4)	5	(2)			
Not working	78	(28)	84	(30)	82	(29)	69	(25)	69	(25)	87	(31)			
No response	14	(5)	8	(3)	13	(5)	8	(3)	10	(4)	10	(4)			

### Identifying emotional experience of manga health communication

The factor analysis’s results are presented in Table 2. Four factors with 15 items emerged. Factor 1: Risk perception conceptualized participants’ concern for their health based on their experience of reading health educational manga. Factor 2: Familiarity was defined as increased positive emotions related to the story and characters in the manga. Factor 3: Satisfaction was identified as a positive psychological state accompanied by cognitive understanding of health information. Factor 4: Realism was regarded as identification of the storyline with the real world and transportation to the character’s emotional state. Sufficient alpha coefficients were observed for all four factors (alpha = 0.81–0.87). Correlation coefficient between factors were relatively high (*r* = 0.59–0.73, *p* < .01). Split-half analysis revealed a strong correlation between the factors (*r* = 0.91, *p* < 0.01). Criterion-related validity confirmed that the correlations among the four factors, and both acceptability (*r* = 0.33, 0.58, 0.48, and 0.52) and usability (*r* = 0.49, 0.60, 0.59, and 0.59) were significant (*p* < .01).

**Table 2.** Structure of the emotional experience of reading health promotion manga.

			Factor			
	*M*	*SD*	1	2	3	4
*F1*: Risk perception (*α* = 0.87)						
I felt a sense of crisis with respect to my health condition.	2.98	0.99	0.97	0.08	−0.10	−0.16
I felt uneasy about my future health condition.	3.00	0.97	0.79	−0.20	0.13	0.08
I realized my neglect of health.	3.05	0.96	0.76	0.07	−0.04	0.03
The speech and behavior of the characters felt as if they were my own.	2.99	0.97	0.51	0.04	0.01	0.30
I imagined my future in which I continued my current lifestyle.	3.22	0.93	0.44	0.25	0.11	0.03
*F2*: Familiarity (*α* = 0.85)						
I felt the content of the story was familiar.	3.28	0.92	0.02	0.71	0.05	0.09
I felt that the characters were likely to exist in reality.	3.39	0.95	−0.02	0.69	−0.18	0.31
I was able to enjoy reading the contents of the health information.	3.29	0.92	0.04	0.65	0.14	−0.01
I felt that the content of the health information was interesting.	3.18	0.89	0.03	0.63	0.28	−0.10
*F3*: Satisfaction (*α* = 0.85)						
I was satisfied to know about health.	3.30	0.84	−0.07	0.10	0.76	0.11
I was happy to know about health.	3.26	0.87	0.06	0.16	0.69	−0.09
It provided the inspiration to think about the current state of my own health situation.	3.28	0.89	0.12	−0.02	0.52	0.28
*F4*: Realism (*α* = 0.81)						
I felt that the recommended content was realistic.	3.46	0.88	−0.03	0.20	0.07	0.62
I empathized with the speech and behavior of the characters.	3.56	0.90	−0.13	0.37	0.02	0.56
I felt that the content is also related to my current self.	3.24	0.92	0.33	0.04	0.00	0.53

**Figure 3.**
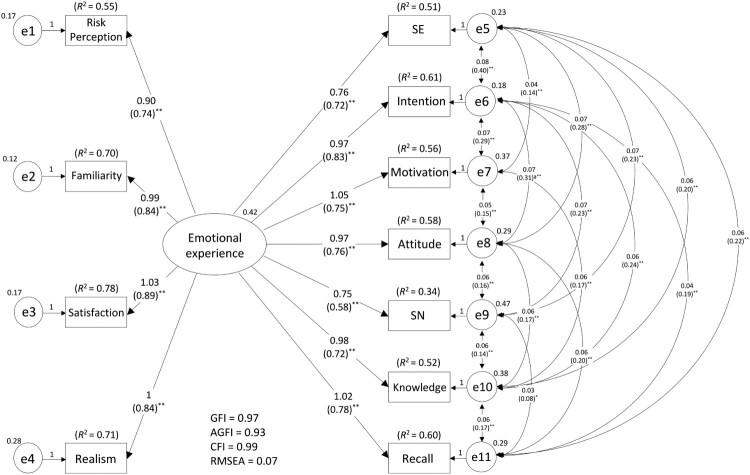
Accepted model of the relationship between the emotional experience of reading health educational manga and readiness of behavior change. Note. Only significant paths were demonstrated. Standardized path coefficients are showed in brackets behind unstandardized path coefficients. Variance is described outside of the latent variables and error variables. Covariance and correlation are shown in brackets with double arrows. SE: self-efficacy, SN: subjective norm. *p<.05, **p<.01.

As depicted in Figure 3, emotional experiences strongly affected the participants’ mediators to engage in behavior change (path coefficient = 0.58–0.83, *p* < .01). The overall model fit index was good for model adaption (GFI = 0.97, AGFI = 0.93, CFI = 0.99, RMSEA = 0.09) and sufficient *R*^2^ values were also obtained (dependent variables of *R*^2^ = 0.34–0.61). Further, the difference in the effect of encouraging psychological mediators depended on which factor was tested. SEM conformed to an acceptable model (GFI = 1.00, AGFI = 0.98, CFI = 1.00, RMSEA = 0.03; Additional file 4).

### Difference of emotional experiences between the six manga and control conditions

The differences in emotional experiences across the six different conditions are presented in Table 3. Overall, the four factors and total scores exhibited significant differences with small effect sizes (Cohen’s *f* = 0.10–0.21, *p* < .01). Post-hoc analysis revealed that the manga conditions were superior to the narrative and general text conditions. Moreover, differences between visual image conditions were found only for satisfaction.

**Table 3.** Difference in emotional experience among the six conditions.

		Intervention			Control			*F*	*df*	*f*	Post-hoc analysis
		SC^a^	HF^b^	RP^c^	Visual image^d^	Narrative^e^	General^f^				
Risk	*M*	3.20	3.21	3.25	2.97	2.87	2.80	3.55**	5	0.10	a-e*
Perception	*SE*	0.07	0.06	0.07	0.07	0.07	0.07				b-e*, f **
	[95%CI]	[3.06, 3.33]	[3.08, 3.33]	[3.11, 3.39]	[2.83, 3.10]	[2.74, 3.01]	[2.66, 2.93]				c-e*, f **
Familiarity	*M*	3.53	3.45	3.51	3.30	3.08	2.84	15.20**	5	0.21	a-e**, f**
	*SE*	0.07	0.06	0.07	0.06	0.06	0.06				b-e**, f**
	[95%CI]	[3.40, 3.66]	[3.33, 3.56]	[3.38, 3.64]	[3.18, 3.43]	[2.96, 3.21]	[2.72, 2.97]				c-e**, f**
Satisfaction	*M*	3.48	3.45	3.53	3.14	3.05	3.03	4.26**	5	0.11	a-d*, e**^,^ f**
	*SE*	0.07	0.06	0.07	0.06	0.06	0.06				b-d*, e**, f**
	[95%CI]	[3.34, 3.61]	[3.33, 3.56]	[3.40, 3.67]	[3.01, 3.26]	[2.92, 3.17]	[2.90, 3.15]				c-d**, e**, f**
Realism	*M*	3.61	3.53	3.69	3.38	3.18	3.13	7.67**	5	0.15	a-e**, f**
	*SE*	0.07	0.06	0.07	0.06	0.06	0.06				b-e*, f**
	[95%CI]	[3.48, 3.73]	[3.42, 3.64]	[3.56, 3.82]	[3.26, 3.51]	[3.06, 3.31]	[3.01, 3.26]				c-e**, f**
Total	*M*	3.42	3.38	3.46	3.17	3.03	2.92	8.36**	5	0.16	a-e**,f**
	*SE*	0.06	0.05	0.06	0.06	0.06	0.06				b-e**, f**
	[95%CI]	[3.31, 3.54]	[3.28, 3.48]	[3.35, 3.58]	[3.06, 3.28]	[2.92, 3.14]	[2.81, 3.03]				c-e**, f**

Note*.* Results of post-hoc analysis are shown as letters corresponding to the superscript letters indicating the names of six conditions; SC: Small change; HF: Habit formation; RP: Relapse prevention. *p<0.05, **p<0.01.

### Differences between primary and secondary outcomes according to the intervention and control conditions

Table 4 presents the results of the primary and secondary outcomes. In the primary outcome analysis, significant differences among the six conditions were observed for both SE and intention (Cohen’s *f* = 0.10, *p* < .01 respectively). However, post-hoc analysis revealed no significant differences among the six conditions in SE. Only marginally significant differences (*p* < .10) were observed in two of the manga conditions (small change and relapse prevention) and the narrative and general text conditions. Behavioral intention was significantly increased in the small change and relapse prevention manga conditions compared with the narrative and general text conditions. Secondary outcome analysis revealed significant differences in motivation, attitude, knowledge, and recall (Cohen’s *f* = 0.10–0.11, *p* < .01). Post-hoc analysis of increases in motivation, attitude, and recall revealed the benefit of using manga in comparison to the narrative and general text conditions. Notably, health educational manga significantly increased the participants’ knowledge in comparison to the visual imagery, narrative, and general text control conditions.

**Table 4.** Psychological readiness change via intervention materials.

		Intervention			Control			*F*	*df*	*f*	Post-hoc analysis
		SC^a^	HF^b^	RP^c^	Visual image^d^	Narrative^e^	General^f^				
SE	*M*	3.44	3.35	3.44	3.29	3.14	3.14	3.50**	5	0.10	
	*SE*	0.06	0.05	0.06	0.06	0.06	0.06				
	[95%CI]	[3.32, 3.56]	[3.24, 3.45]	[3.32, 3.56]	[3.18,3.41]	[3.03, 3.26]	[3.02, 3.25]				
Intention	*M*	3.43	3.39	3.51	3.22	3.08	3.14	3.17**	5	0.10	a-e*
	*SE*	0.07	0.06	0.07	0.06	0.06	0.06				
	[95%CI]	[3.30, 3.57]	[3.27, 3.51]	[3.38, 3.64]	[3.09, 3.34]	[2.96, 3.21]	[3.01, 3.27]				c-e**, f*
Motivation	*M*	3.36	3.33	3.50	3.16	3.00	3.13	3.05**	5	0.10	
	*SE*	0.08	0.07	0.08	0.08	0.08	0.08				
	[95%CI]	[3.20, 3.52]	[3.19, 3.47]	[3.33, 3.66]	[3.01, 3.32]	[2.85, 3.16]	[2.97, 3.28]				c-e**
Attitude	*M*	3.53	3.42	3.57	3.25	3.08	3.12	3.85**	5	0.11	a-e**, a-f*
	*SE*	0.07	0.07	0.08	0.07	0.07	0.07				
	[95%CI]	[3.39, 3.68]	[3.29, 3.55]	[3.42, 3.72]	[3.11, 3.39]	[2.94, 3.22]	[2.97, 3.26]				c-e**, f**
SN	*M*	3.29	3.19	3.28	3.14	3.01	3.01	1.77	5	0.07	
	*SE*	0.08	0.07	0.08	0.07	0.07	0.07				
	[95%CI]	[3.14, 3.44]	[3.06, 3.32]	[3.13, 3.43]	[2.99, 3.28]	[2.86, 3.15]	[2.87, 3.16]				
Knowledge	*M*	3.58	3.43	3.60	3.12	3.01	3.14	4.23**	5	0.11	a-d*, e**, f*
	*SE*	0.08	0.07	0.08	0.08	0.08	0.08				b-e*
	[95%CI]	[3.42, 3.74]	[3.29, 3.56]	[3.45, 3.76]	[2.97, 3.27]	[2.86, 3.16]	[2.99, 3.29]				c-d**, e**, f*
Recall	*M*	3.49	3.40	3.53	3.26	3.11	3.14	3.20**	5	0.10	a-e*
	*SE*	0.07	0.06	0.07	0.07	0.07	0.07				
	[95%CI]	[3.34, 3.63]	[3.27, 3.53]	[3.39, 3.68]	[3.13, 3.40]	[2.97, 3.24]	[3.00, 3.27]				c-e*, f*

Note. Results of post-hoc analysis are shown as letters corresponding to the superscript letters indicating the names of the six conditions; SC: Small change; HF: Habit formation; RP: Relapse prevention; SE: self-efficacy; SN: subjective norm. *p<0.05, **p<0.01.

## Discussion

This study conducted a PAHDEM trial to identify the emotional experiences of reading educational manga that focuses on promoting physical activity and healthy diet; to understand the effect of the psychological mediators for behavior change; and to confirm the differences in intervention effects to increase the factors of the psychological mediators of behavior change among health educational manga and traditional intervention materials. Four emotional factors emerged: risk perception, familiarity, satisfaction, and realism. Additionally, the results revealed that the total scores for the emotional experiences and four independent factors strongly predicted psychological mediators for behavior change. Further, the manga conditions were superior in terms of experienced risk perception, familiarity, and realism in comparison to the narrative and general text-based conditions. Satisfaction was greater in the manga conditions than the three control groups. The benefits of using manga in comparison to the narrative and general text conditions were identified; these included increased intention, motivation, attitude, knowledge, and recall. A significant difference between the manga and visual image conditions was only confirmed for increased knowledge. Possible explanations for the findings are discussed subsequently.

Studies that have examined the contribution of emotional factors and effects of entertainment education interventions have revealed positive effects of promoting behavior change by encouraging empathy with the storyline and characters (Quintero Johnson et al., 2013). The current findings revealed similar factors, such as narrative transportation, identification of the character, and perceived reality (Quintero Johnson et al., 2013; van Leeuwen et al., 2013), which were significant predictors of psychological mediators for behavior change. However, the present results revealed that reading health educational manga induced more complex emotional experiences and affected participants’ readiness for healthy behavior changes. Different dimensions of perceived benefit-loss to engage or not engage in health behavior were identified. A significant finding was that the effect of risk perception including fear of continuing current unhealthy lifestyle habits also predicted psychological mediators. The current findings may be related to the framing effect. Studies that explored the framing effect (Tversky & Kahneman, 1981) found that emotional experiences such as empowerment or perceived fear from health messaging affected decision-making about health-related behavior. The studies on framing revealed that health behavior promotion, including physical activity and a healthy diet, was facilitated by gain-framed messaging, enabling participants to empathize with the benefits of engaging in health behavior change (Gallagher & Updegraff, 2012; O’Keefe & Jensen, 2007). The theory of psychological reactance posited that individuals react with unpleasant responses when exposed to messages with negative, risk, and fear appraisal connotations (Brehm & Brehm, 1981). However, the findings of the present study indicated emotional experience via loss-framed messaging also encourages psychological mediators. Several studies reported that controversial insistence, which perceives that seriousness may encourage preventive behavior. The well-established Health Belief Model of Becker, Maiman, Kirscht, Haefner, and Drachman (1977) noted that perceived threat of disease may predict engagement in health protection behavior. In addition, in the entertainment education context, Bieri, Yuan, et al. (2013) noted the risk factor of worm infection and implementation of cartoon videos display increased preventive effects against infection (Bieri, Gray, et al., 2013). One possible explanation is that entertainment education includes manga-style instruction, which promotes the communication of serious messages without psychological reactance (Branscum & Sharma, 2009). Thus, further studies are required to consider the differences and effects of increasing risk perception using manga and other health educational media and their contribution in predicting changes in physical activity and healthy diet behavior.

In addition, the present study confirmed the reliability and validity of the inventory for emotional experience from reading health educational manga according to internal consistency (i.e. calculations of alpha values and split-half reliability coefficient), criterion-related validity (i.e. correlation analysis with an acceptability–usability scale), and part of construct validity (i.e. SEM). In this regard, further study was required to confirm test–retest reliability, content validity, cross-cultural validity, responsiveness, and interpretability following the COnsensus-based Standards for the selection of health Measurement Instruments (COSMIN) framework, which is widely used to improve the quality of the design of studies on instruments for health-related measurements (COSMIN, 2021; Mokkink et al., 2010).

Our results revealed significant differences between the manga intervention conditions and the narrative and general text control conditions in relation to the effects on emotional experiences and outcomes. Further, this study evinced that according to the psychological burden of text reading (Glanz & Rudd, 1990), text-based information had a smaller intervention effect even when a narrative story-telling approach was compared to the manga intervention. However, few differences between the manga and visual image-based information conditions were found. Although the effects of health educational manga may be ascribed to the effects of visual image stimuli (Houts et al., 2006; Scott et al., 2016), the study findings revealed that manga-based interventions induced satisfaction, which led to increased knowledge in comparison to the visual image control condition. Many educational text and materials have utilized manga as an effective learning tool (Farinella, 2018; Hosler & Boomer, 2011). The controversial results have included whether health education manga is superior for obtaining health-related knowledge in the health education setting. Several studies have also reported that health educational manga contributed to increased knowledge (Dworkin et al., 2013; el-Setouhy & Rio, 2003; Yuan et al., 2000). Conversely, no significant differences have been observed between other types of materials or education (Baranowski et al., 2002; Leung et al., 2014, 2017). Therefore, less is known about the mechanism underlying the facilitation of comprehension of health information using manga. McNicol (2017) has suggested that the sense of reassurance (i.e. positive mood state) via reading health educational manga encourages the recall of health-related information. In addition, the result of the present study has indicated that employing visual image information in health care and medical settings may be disadvantaged because sometimes individuals interpret messaging as ambiguous (Houts et al., 2006). Because visual image stimuli operate on visual sensory channels, it may be difficult to induce deep thinking and cognition. In accordance with the current results, it is believed that manga-based approaches may enable the combination of cognitive understanding based on narrative story-telling to provide practical knowledge in a general lifestyle context as well as the effects of visual image stimuli on sensory understanding and reduced psychological burden. Nevertheless, the overall small effect size observed in the results of the current study is consistent with those of previous randomized controlled trials (Junhasavasdikul et al., 2017; Kovacs et al., 2011).

Although this study was a well-controlled low bias risk trial, several potential limitations should be examined. First, although the study investigated educational backgrounds to consider the differences in the comprehensiveness of intervention materials, it did not assess preference and interest for manga or other popular media. These variables may influence the results through emotional arousal. Second, the participants were only assessed after exposure to an intervention stimulus, i.e. post-only assessment due to grant concerns. Therefore, changes in psychological mediators between baseline and follow-up data could not be examined. Third, because the longitudinal effects of manga-based intervention could not be examined, it is unclear whether the increase in psychological mediators was short- or long-term. Fourth, secondary outcomes were measured with a single questionnaire item to minimize the length of the questionnaire to increase compliance. Therefore, although the present study did not have any missing values, the assessment for the secondary outcomes did not possess sufficient reliability and validity. Fifth, the participants’ emotional experiences of reading manga materials were examined. The underlying concepts and predictive effects on behavior change readiness may be similar when other entertainment educational intervention media is employed. Further studies are required to confirm the factor structure of the current study and to assess the mediating effects of entertainment education other than manga-based materials. Sixth, the present study did not consider question–behavior effects. Previous studies reported the potential of the contents of the presented questionnaire effects in influencing decision-making to practice health behaviors (Doyle et al., 2019; Wilding et al., 2016). As defined in the present study, emotional experience pertains to the positive and negative aspects of emotion, which may have induced the question–behavior effects. Seventh, Dima (2018) proposed a six-step protocol for constructing psychological scales. Although the present study covered the overall protocol, testing via item response theory was not conducted according to the technical limitation of the protocol. Thus, further tests are required to verify reliability and validity.

Finally, although the use of health educational manga was found to be effective for promoting behavior changes in comparison to visual imagery, narrative texts, and general information, other entertainment media that incorporates sound information such as animation and sound- or voice-based materials was not considered.

## Conclusion

The study findings demonstrated the advantages of using a manga-based approach in the health educational context in accordance with psychological effects. The emotional experiences of reading health educational manga combined sensory and cognitive methods for facilitating psychological mediators change. The current findings help extend current understanding of why health educational manga is an effective strategy for health communication. Further studies from other scientific disciplines including brain research and other natural sciences are required to elucidate the mechanisms underlying the effectiveness of health educational manga. Further, it is recommended that future studies explore whether emotional experiences also contribute to behavior change by employing other entertainment media such as games (Koivisto & Hamari, 2019), animation (Bieri, Gray et al., 2013), and drama (Perry, Zauner, Oakes, Taylor, & Bishop, 2002). In the Japanese context, manga is not only an entertainment medium but also a significant cultural form (Ito, 2005). Understanding of the acceptability and usability of educational manga is increasing internationally (Lo et al., 2019). Although the present study has several limitations, expanding evidence-based health communication and health promotion practice by utilizing educational manga may contribute to the development of health and well-being in the international setting.

## Supplementary Material

Supplemental Material
