# Supplementary material for: Emotional experiences of reading health educational manga encouraging behavioral changes: a non-randomized controlled trial
Source: Health Psychol Behav Med. 2021 Apr 30;9(1):398–421. doi: 10.1080/21642850.2021.1921583 (PMC8159205; doi:10.1080/21642850.2021.1921583)
Supplement: Supplemental Material [file RHPB_A_1921583_SM1419.zip › Additional file 2.docx]

**­Additional file 2. Intervention and control materials**

**I. Intervention materials**

**1. Manga #1 Small change (with English version dialogue and topics)**


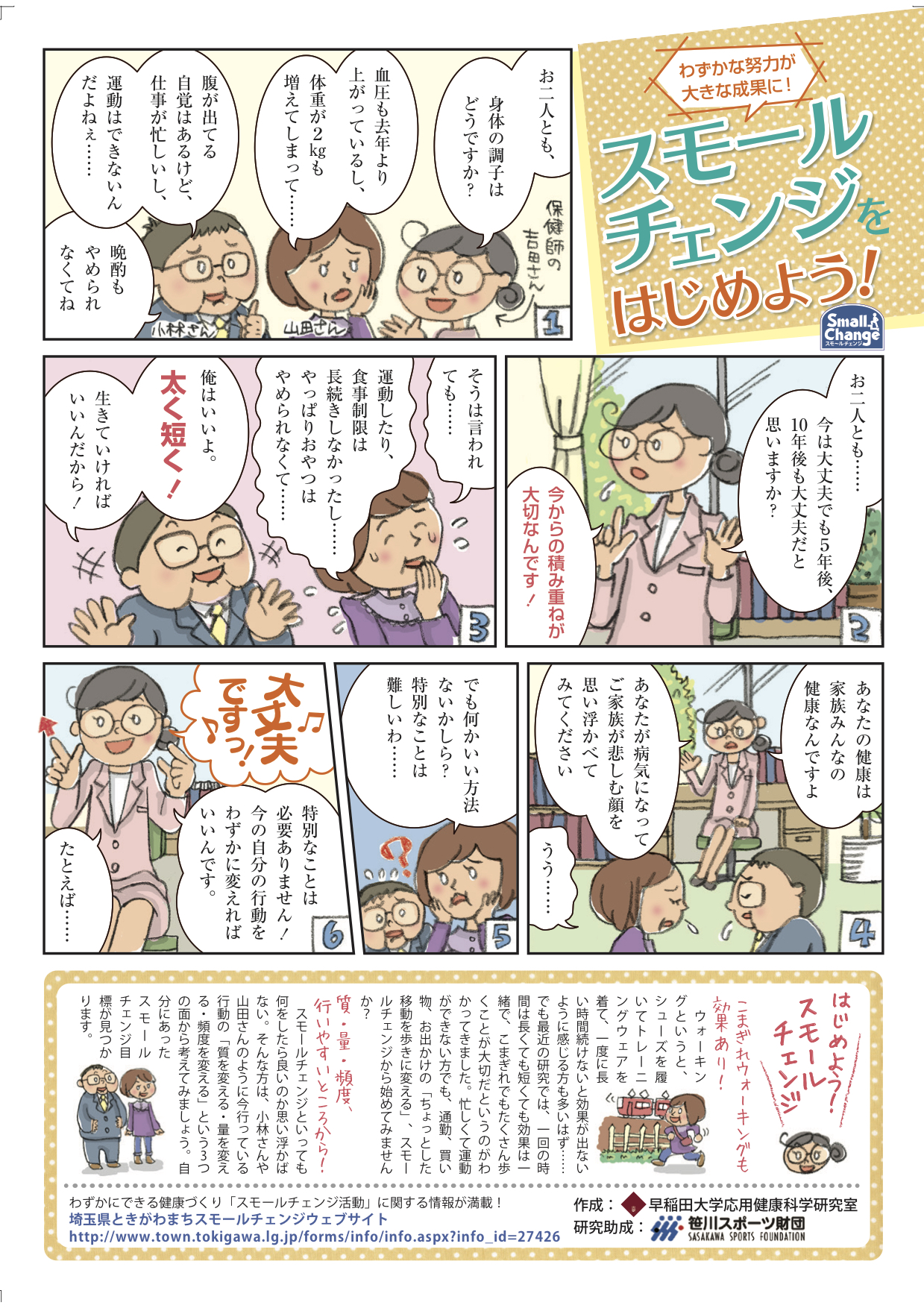


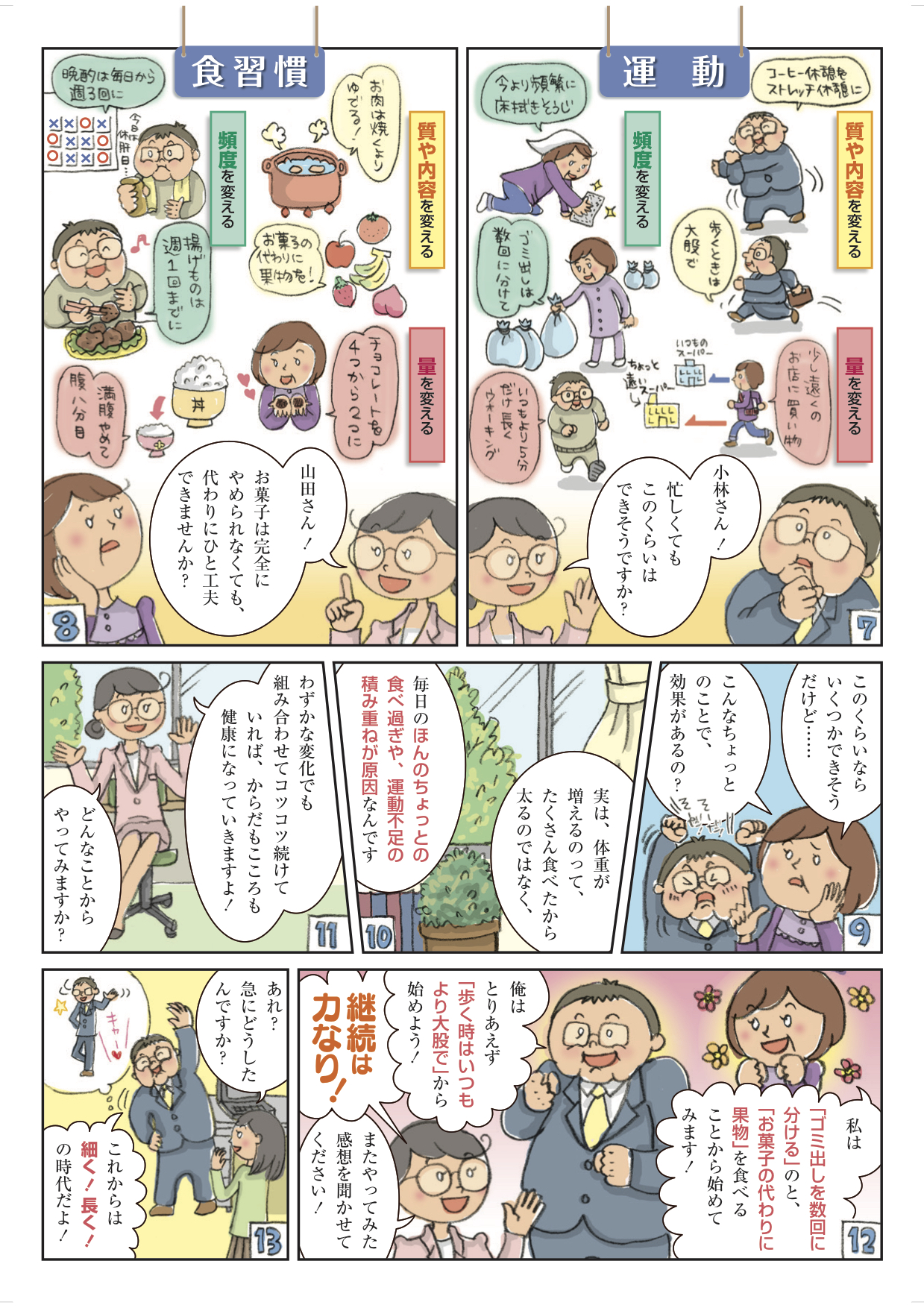


| Frame | Dialogue |
| --- | --- |
| 1 | *PHN Yoshida*: How is your health, both of you? |
|  | *Mrs. Yamada:* Blood pressure has also increased since last year and weight has also increased by 2 kilograms… |
|  | *Mr. Kobayashi*: I am aware that I have a paunch but I am busy with work and cannot exercise…and I have also not been able to stop drinking at dinner. |
| 2 | *PHN Yoshida*: Both of you…at present all is fine but do you think five years, ten years from now too all will be fine? It is important to make efforts from now onwards! |
| 3 | *Mrs. Yamada*: Even then …I have not been able to continue exercising and restricting my diet for a long time…also, I cannot really give up sweets… |
|  | *Mr. Kobayashi*: I am fine. It’s all right to live short if I can do what I want to do! |
| 4 | *PHN Yoshida*: Your health is the health of all the members of your family. Please think of all the sad faces of your family members if you fall ill. |
|  | *Mr. Kobayashi & Mrs. Yamada*: Hmm… |
| 5 | *Mrs. Yamada*: But isn’t there a good way of doing it? It is difficult to do something out of the way… |
| 6 | *PHN Yoshida*: Don’t worry! There is no need to use anything special! All you need to do is just change your everyday actions a little. For example… |
| 7 | *PHN Yoshida*: Mr. Kobayashi! Even though you are busy, will you be able to manage this much? |
|  | [*Physical activity small change*] *Change the nature or content*:;; *Changing the quantity*:; walk for 5 minutes longer than usual; *Change the frequency*: mop the floor clean more frequently than at present; divide the trash and put it out more often. |
| 8 | *PHN Yoshida*: Ms. Yamada! There is no need to stop eating sweets altogether, but do you think you can come up with a way of reducing intake of sweets? |
|  | [*Healthy eating small change*] *Change the nature or content*: meat is better boiled rather than grilled; fruits instead of sweets; *Changing the quantity*: reduce chocolate to two pieces from four pieces; eat moderately and never fill your stomach; *Change the frequency*: reduce drinking at dinner from every day to three times a week; eat deep-fried food only once a week. |
| 9 | *Mrs. Yamada*: I think I may be able to manage this much…Will these small things be effective? |
| 10 | *PHN Yoshida*: Actually, weight does not increase because one has eaten too much. The cause is the piling up of the little bit of overeating and lack of exercise every day. |
| 11 | *PHN Yoshida*: Even small changes when continued consistently make both the body and mind healthy! What would you like to begin with? |
| 12 | *Mrs. Yamada*: I would like to divide the garbage and increase the number of times I go to put out the trash and start eating fruits instead of sweets! |
|  | *Mr. Kobayashi*: For the time being, I would like to start with “walking in longer strides that usual”! |
|  | *PHN Yoshida*: Perseverance will accomplish all things! Do let us know about your experiences! |
| 13 | *Co-worker*: What’s this? What’s happening all of a sudden? |
|  | *Mr. Kobayashi*: The future is all about having a long, frugal life! |

| Topics | Description |
| --- | --- |
| Walking bit by bit is also effective! | Many people should feel that putting on shoes and training wear and going for a walk is not effective unless you take a long walk… However, recent research has shown that walking a lot, even a bit at a time, is important. This is due to the cumulative effect of both short and long walks. If you are someone who is busy and cannot exercise, how about starting with small changes? Such as, “changing brief trips into walks.” |
| Start from the easy things, such as quality, quantity and frequency! | It is difficult to come up with small changes. People who struggle to come up with something, like Kobayashi and Ms. Yamada, should start by considering their current behavior from three perspectives: “changing quality,” “changing quantity” and “changing frequency.” That way, you can identify a goal of small changes that suit you. |

**2. Manga #2 Habit formation (with English version dialogue and topics)**


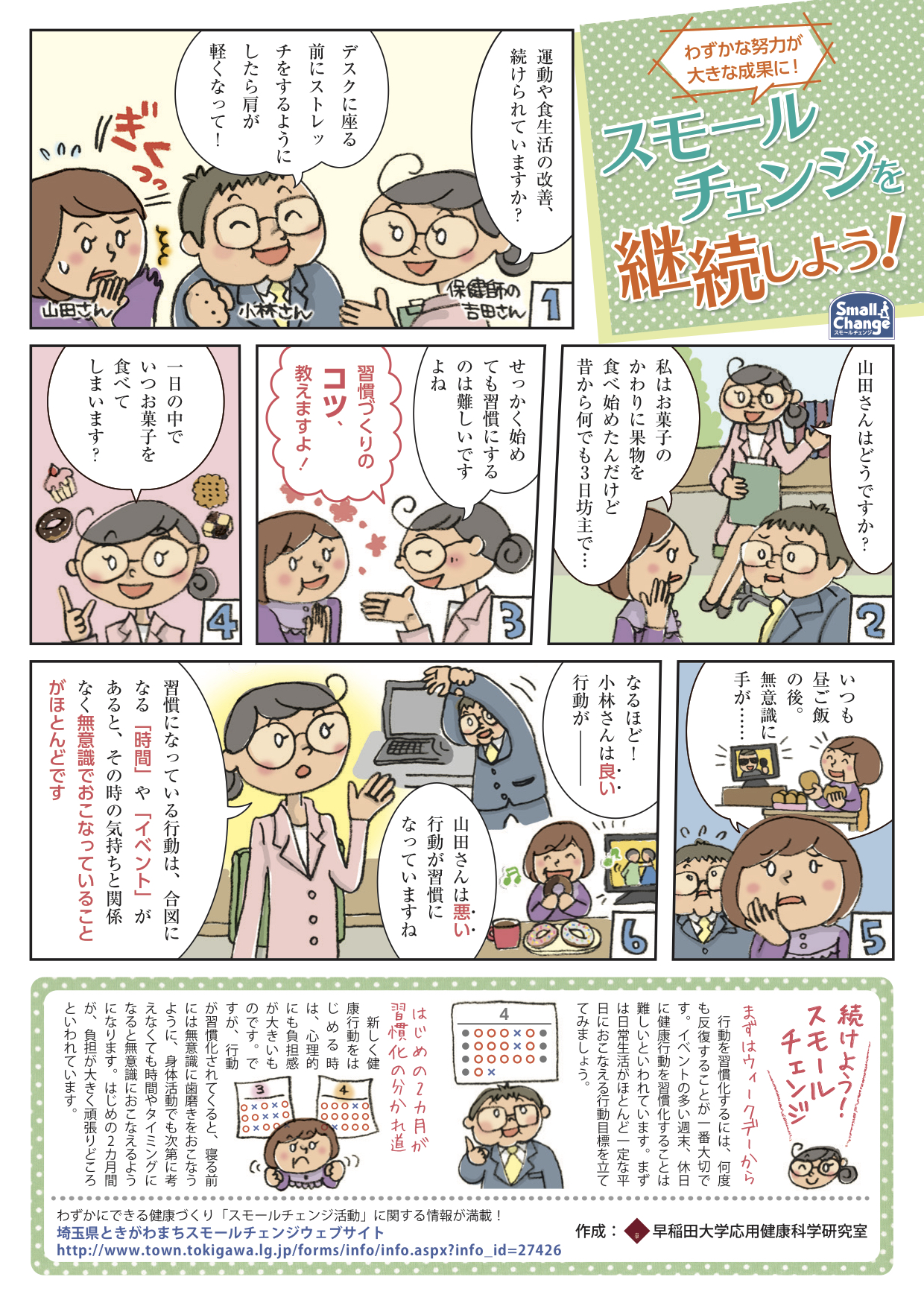


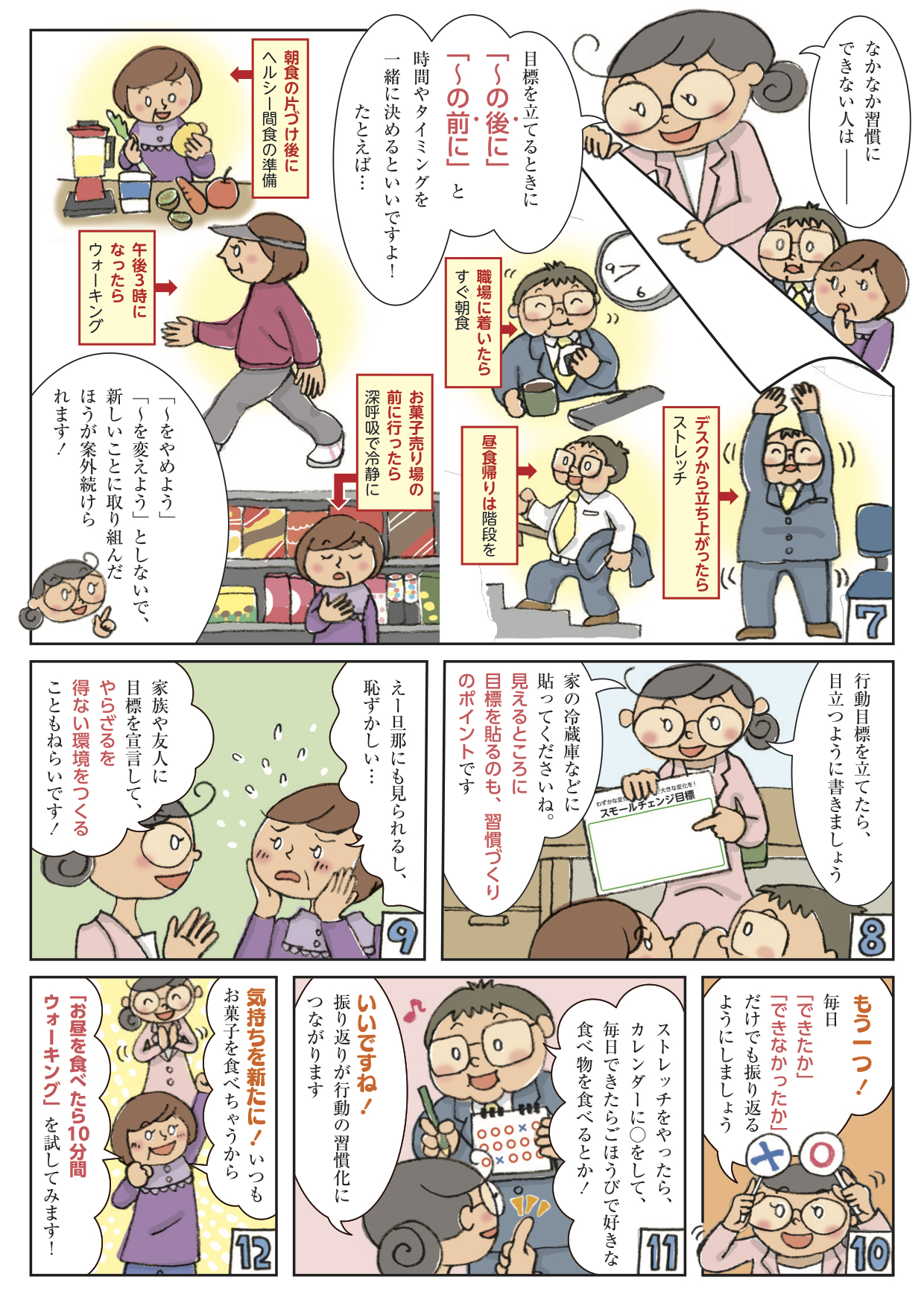


| Frame | Dialogue |
| --- | --- |
| 1 | *PHN Yoshida*: Have you continued to improve your exercise and dietary lifestyle? |
|  | *Mr. Kobayashi*: My shoulders are less stiff now that I do stretching before sitting at my desk! |
|  | *Mrs. Yamada:* Wow. |
| 2 | *PHN Yoshida*: How about you, Ms. Yamada? |
|  | *Mrs. Yamada:* I have started eating fruit instead of sweets, but I have always lacked perseverance… |
| 3 | *PHN Yoshida*: It’s difficult to make something a habit despite finally starting it.  Let’s learn tricks to form habits! |
| 4 | *PHN Yoshida*: When do you end up eating sweets during the day? |
| 5 | *Mrs. Yamada:* Always after lunch. I unconsciously reach for them… |
| 6 | *PHN Yoshida*: I see! Kobayashi is developing a good habit, while Ms. Yamada is developing a bad habit.  Habitual behavior mostly occurs unconsciously in response to a “time” or “event” regardless of one’s feelings at that time. |
| 7 | *PHN Yoshida*: When people who struggle to develop habits set goals, they should decide on the period and timing together, such as “after X” or “before Y”! For example…  [*Example of behavior for Mr. Kobayashi*] (1) eat breakfast as soon as you arrive at work, (2) do stretching once you stand up from your desk, (3) take the stairs when returning from lunch  [*Example of behavior for Mrs. Yamada*] (1) prepare a healthy snack after clearing away your breakfast, (2) go for a walk at 3 in the afternoon, (3) calm yourself with deep breaths when you pass in front of sweet selling area in a shop  *PHN Yoshida*: You can actually keep habits going by taking on new challenges without thinking, “I should stop X” or “I should change Y”! |
| 8 | *PHN Yoshida*: Let’s list our behavioral goals in an noticeable place.  Please attach your list to the fridge or something else at home. Putting up your list of goals in a visible place can help build habits. |
| 9 | *Mrs. Yamada:* Um, my husband would also see the list. That would be embarrassing…  *PHN Yoshida*: Announce your goals to your family and friends. That way you can also establish an environment that forces you to keep to your goals! |
| 10 | *PHN Yoshida*: One more thing! Let’s try to look back on our achievements by simply establishing if we “succeeded at” or “did not succeed at” our daily goals. |
| 11 | *Mr. Kobayashi*: Like drawing a circle on the calendar if you did stretching and rewarding yourself with your favorite foods if you managed to stretch every day!  *PHN Yoshida*: Yes, that’s good! Reflection is linked to forming habits. |
| 12 | *Mrs. Yamada:* It’s about changing the way you think! Because I tend to eat sweets, I’ll try “taking a 10-minute walk after lunch”! |

| Topic | Description |
| --- | --- |
| Start with weekdays | Repetition is the most important part of forming habits. It is said to be difficult to form health habits on weekends and holidays which might have many events. Start by establishing behavioral goals that can be accomplished on weekdays where your lifestyle is mostly fixed. |
| The first 2 months are where habits are made or broken | The time at the start of new forming health habits is very tough psychologically. However, when a habit begins to form, you start to unconsciously and gradually perform this physical activity at the right period and timing, such as unconsciously brushing your teeth before bed. The first 2 months are said to be stressful and require perseverance. |

**3. Manga #3 Relapse prevention (with English version dialogue and topics)**


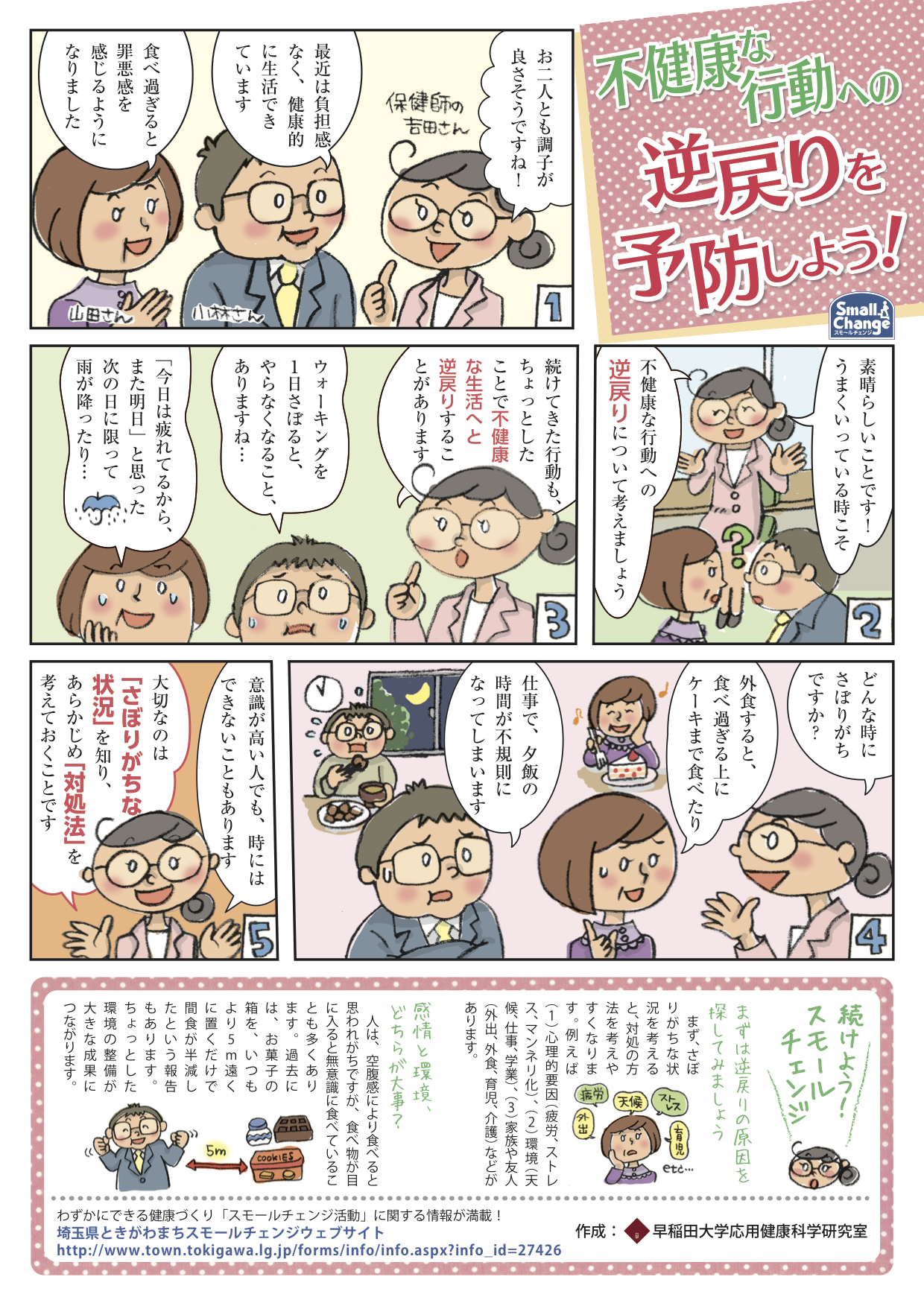


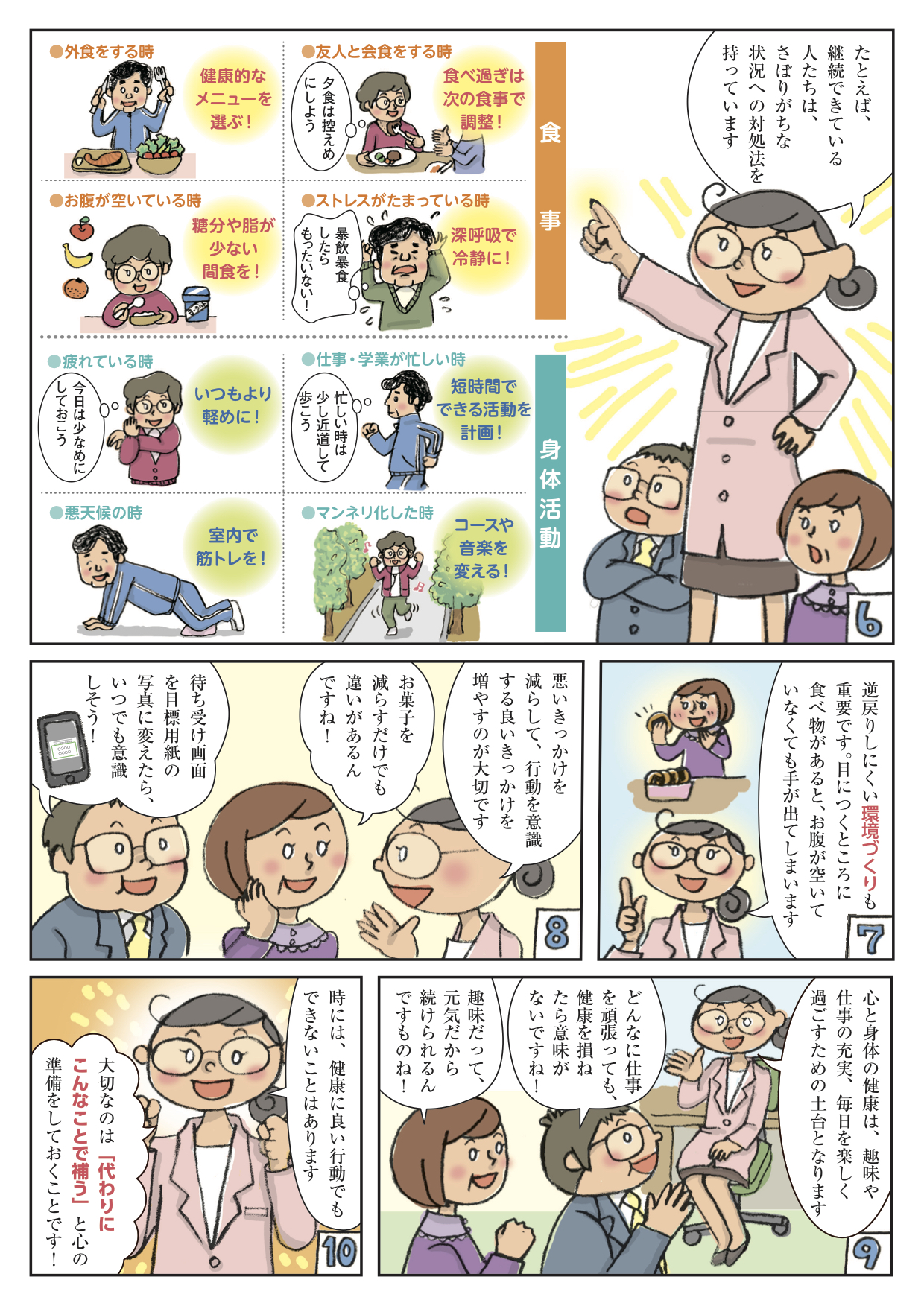


| Frame | Dialogue |
| --- | --- |
| 1 | *PHN Yoshida*: You both seem like you are in a good shape!  *Mr. Kobayashi*: Recently, we haven’t had anything burdensome to bear and we’ve been able to lead a healthy lifestyle.  *Mrs. Yamada:* We have started feeling guilty if we eat too much. |
| 2 | *PHN Yoshida*: That’s wonderful! Because things are going so well, let’s think about regression to unhealthy habits. |
| 3 | *PHN Yoshida*: Sometimes, even when you have continued healthy behaviors, you can revert to unhealthy habits from one small thing.  *Mr. Kobayashi*: There are some days when you don’t walk for even one day…  *Mrs. Yamada* You think, “I’m tired today, so I’ll walk tomorrow,” and then the next day it rains… |
| 4 | *PHN Yoshida*: When are you likely to slack off?  *Mrs. Yamada* When I eat out, in addition to overeating, I eat cake.  *Mr. Kobayashi*: At work, when dinner time becomes irregular. |
| 5 | *PHN Yoshida*: Even people who are highly conscious are sometimes not able to do things.  What’s important is to know the “situations where you tend to fall off,” and to think of “coping methods” ahead of time. |
| 6 | *PHN Yoshida*: For example, people who are able to continue have coping methods for situations where they slack off.  [Health eating relapse prevention] (1) In the case of dining out → choose from a healthy menu (2) When you’re hungry → take snacks with low sugar and fat (3) In the case of eating with friends → if you overeat, adjust the following meal (cut back on dinner), (4) For stressful times → Take deep breaths and stay calm (overeating and overdrinking does not do anything!)  [Physical activity relapse prevention] (1) When you’re tired → Keep it more moderate than usual (just do a little today), (2) In the case of bad weather → Do muscle training indoors, (3) When busy at work/school → Plan activities you can do in short time periods (when things are busy, take a short walk), (4) In the case of habits → Change course and music. |
| 7 | *PHN Yoshida*: It is also important to create an environment that makes it difficult to relapse. If food is within eyesight, people reach for it even when they are not hungry. |
| 8 | *PHN Yoshida*: It is important to decrease bad opportunities and increase good opportunities with awareness of behavior.  *Mrs. Yamada* Even hiding sweets makes a difference!  *Mr. Kobayashi*: If I change the standby screen to a photo of my goals sheet, I will always be aware! |
| 9 | *PHN Yoshida*: Mental and physical health becomes the foundation for fulfilling your hobbies and work and enjoying every day.  *Mr. Kobayashi*: No matter how hard you work, if you lose your health, it will be meaningless!  *Mrs. Yamada:* Even for hobbies, if you are in good health, you will be able to continue them! |
| 10 | *PHN Yoshida*: Sometimes, you are unable to practice behavior that is good for your health.  *PHN Yoshida*: The important thing is to say, “Instead, I will make up for it with this,” and prepare your mindset! |

| Topic | Description |
| --- | --- |
| First, let’s investigate causes for reversion. | First, when you think about the situations where you tend to fall off, it becomes easy to consider coping mechanisms. For example, (1) psychological causes (fatigue, stress, habits), (2) environment (weather, work, school work), (3) family and friends (going out, dining out, childcare, nursing care), etc. |
| Feelings or environment? Which is important? | We tend to think that people eat because they feel hungry, but often they eat without thinking when they see food. It has been previously reported that snacking is cut in half simply by placing a candy box 5m farther away than usual. Slight environment maintenance leads to great results. |

**Ⅱ. Control stimulus**

**1. Control #1: Visual image condition**


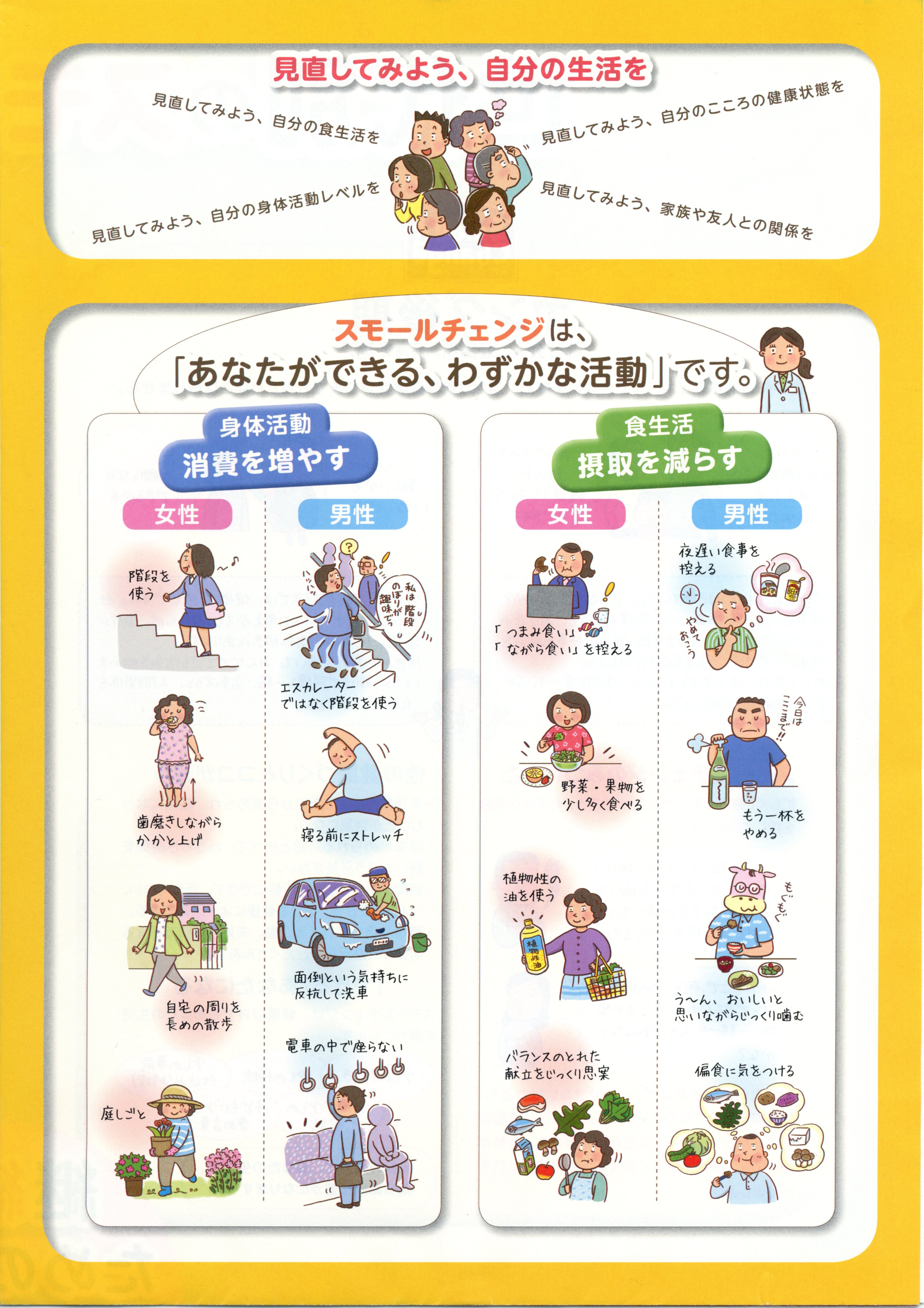


(English translation)

| Let’s reassess your daily life | |
| --- | --- |
| Let’s reassess your eating habits | Let’s reassess your mental health condition |
| Let’s reassess your daily physical activity level | Let’s reassess your relationship with your family and friends |

| Small change is “a small lifestyle modification that you can do easily” | | | |
| --- | --- | --- | --- |
| Physical activity Increase energy output | | Eating habits Decrease amount of intake | |
| Women | Men | Women | Men |
| Using stairs | Using stairs instead of an escalator | Quitting “Bite eating” and “While eating” | Avoiding midnight dinners |
| Doing “up end down heel” during tooth brushing | Stretching before going to bed | Eating more fruit and vegetables | Quitting alcohol consumption |
| Taking extended walks in the neighborhood | Getting a car washing without bothering | Using vegetal oil | Chewing thoughtfully |
| Gardening | Standing on the train | Considering the balance of nutrients | Correcting an unbalanced diet |

**2. Control #2: Narrative condition**


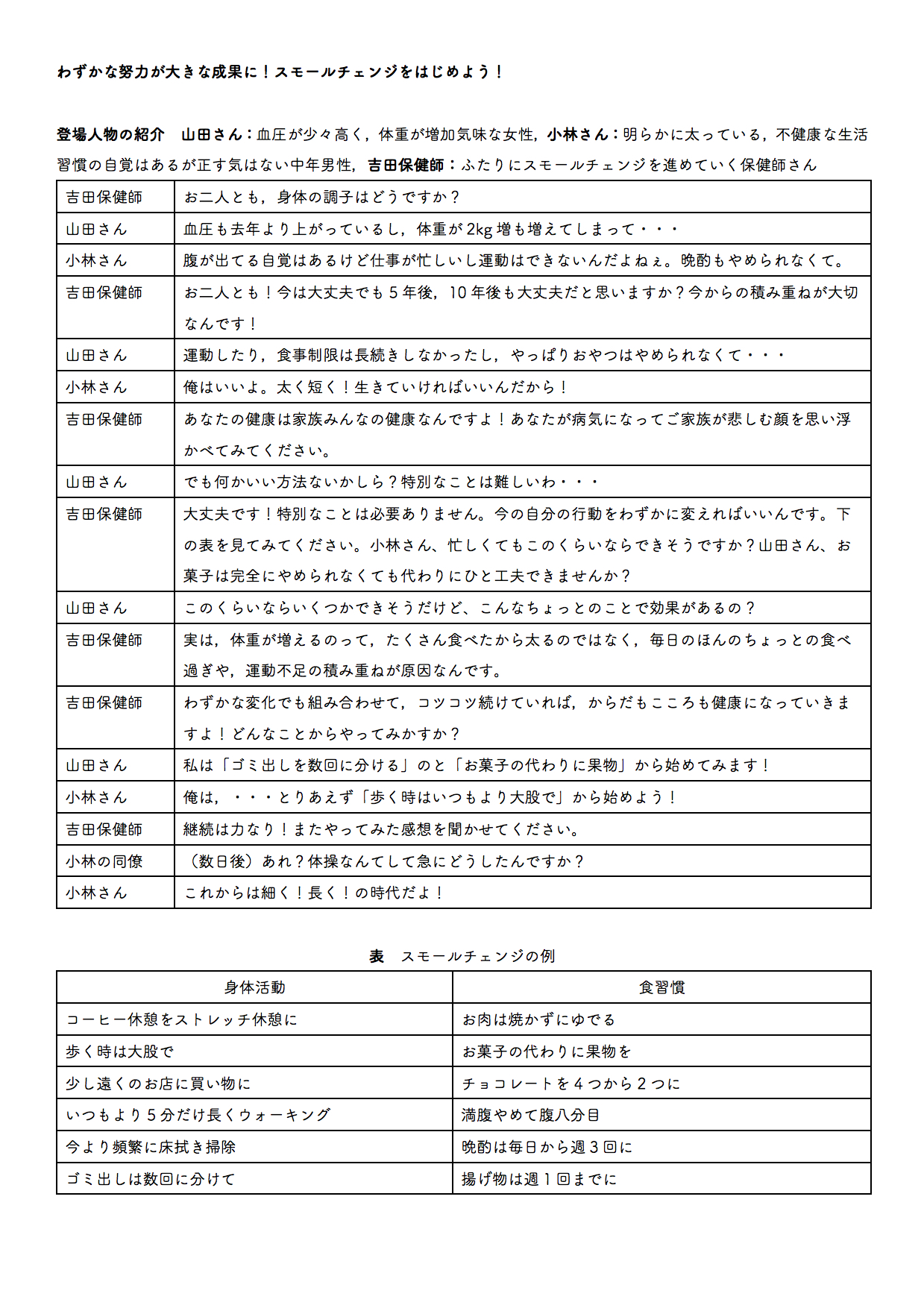


(English translation)

Slight effort leads big achievement! Let’s start small change

Introduce characters: *Mrs. Yamada* concerns blood pressure and recently weight gain; *Mr. Kobayashi* is obviously over weight men and he notice but no intention to improve; *Public health nurse* (*PHN) Yoshida* recommends small life style change to them

| *PHN Yoshida* | How is your health, both of you? |
| --- | --- |
| *Mrs. Yamada* | Blood pressure has also increased since last year and weight has also increased by 2 kilograms… |
| *Mr. Kobayashi* | I am aware that I have a paunch but I am busy with work and cannot exercise…and I have also not been able to stop drinking at dinner. |
| *PHN Yoshida* | Both of you…at present all is fine but do you think five years, ten years from now too all will be fine? It is important to make efforts from now onwards! |
| *Mrs. Yamada* | Even then …I have not been able to continue exercising and restricting my diet for a long time…also, I cannot really give up sweets… |
| *Mr. Kobayashi* | I am fine. It’s all right to live short if I can do what I want to do! |
| *PHN Yoshida* | Your health is the health of all the members of your family. Please think of all the sad faces of your family members if you fall ill. |
| *Mrs. Yamada* | But isn’t there a good way of doing it? It is difficult to do something out of the way… |
| *PHN Yoshida* | Don’t worry! There is no need to use anything special! All you need to do is just change your everyday actions a little. For example, please see bellow table. Mr. Kobayashi! Even though you are busy, will you be able to manage this much? Ms. Yamada! There is no need to stop eating sweets altogether, but do you think you can come up with a way of reducing intake of sweets? |
| *Mrs. Yamada* | I think I may be able to manage this much…Will these small things be effective? |
| *PHN Yoshida* | Actually, weight does not increase because one has eaten too much. The cause is the piling up of the little bit of overeating and lack of exercise every day. |
| *PHN Yoshida* | Even small changes when continued consistently make both the body and mind healthy! What would you like to begin with? |
| *Mrs. Yamada* | I would like to divide the garbage and increase the number of times I go to put out the trash and start eating fruits instead of sweets! |
| *Mr. Kobayashi* | For the time being, I would like to start with “walking in longer strides that usual”! |
| *PHN Yoshida* | Perseverance will accomplish all things! Do let us know about your experiences! |
| *Kobayashi’s co-worker* | What’s this? What’s happening all of a sudden? |
| *Mr. Kobayashi* | The future is all about having a long, frugal life! |

Table Examples of small lifestyle change

| Physical activity | Eating behavior |
| --- | --- |
| Coffee break into a stretch break | Meat is better boiled rather than grilled |
| Walk in long strides | Fruits instead of sweets |
| Go shopping to a store that is a little further away | Reduce chocolate to two pieces from four pieces |
| Walk for 5 minutes longer than usual | Eat moderately and never fill your stomach |
| Mop the floor clean more frequently than at present | Educe drinking at dinner from every day to three times a week |
| Divide the trash and put it out more often | Eat deep-fried food only once a week |

**3. Control #3: General information**


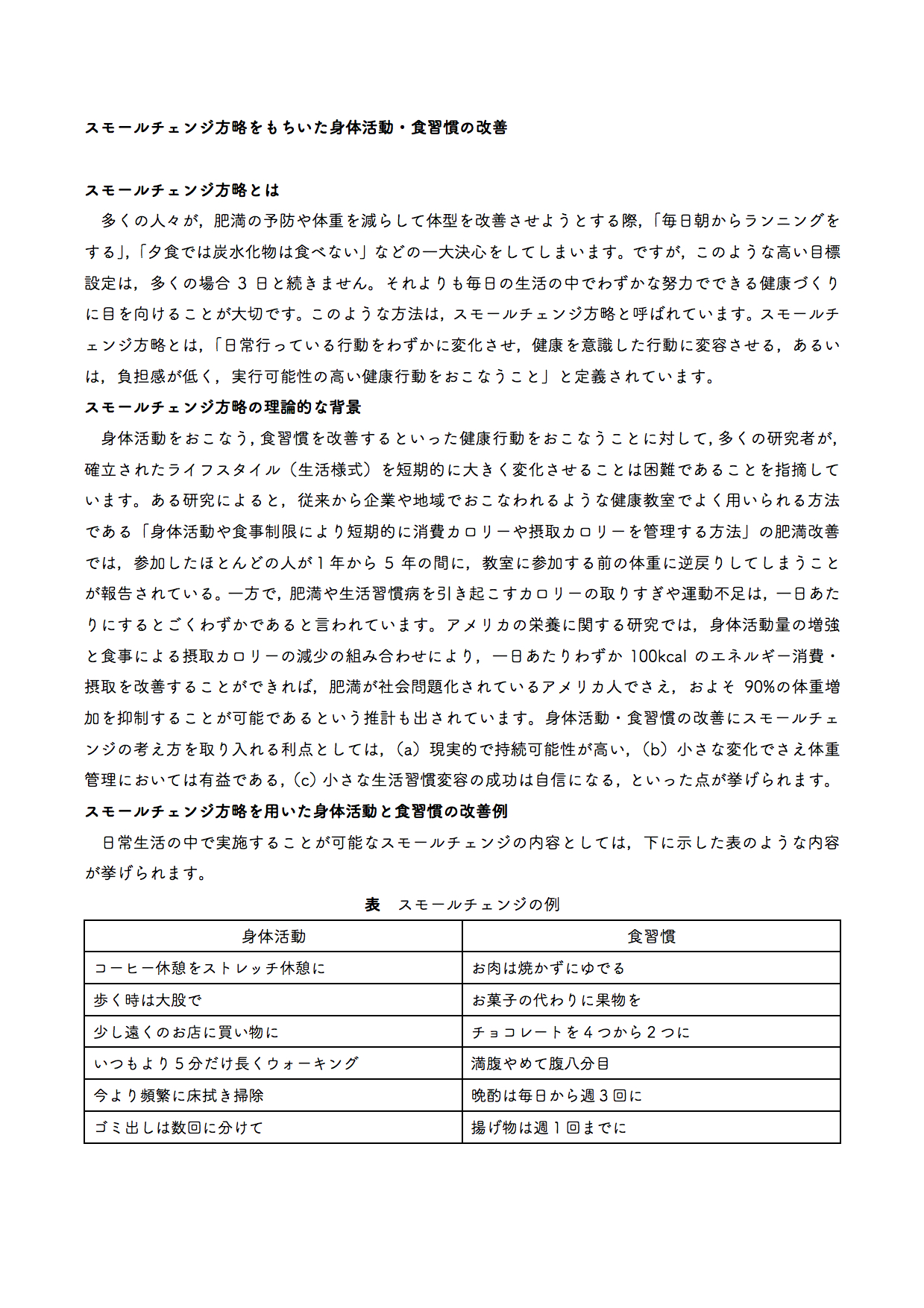


(English translation)

**Improving physical activity and eating habits using the small change strategy**

**: What is the small change strategy**

Many people set high goals for themselves to prevent obesity and for weigh reduction, such as “I will run every morning” or “I will eat no carbohydrates at dinner”. However, such goal setting often cannot maintained for even 3 days. Rather than setting such lofty goals, a focus on achievable goal setting in daily living is more important. Such a strategy is called the small change strategy. The small change strategy is defined as “making small lifestyle changes toward health promotion in daily living, or promoting a low-psychological burden and high feasibility of healthy behavior”.

**Theoretical background for the small change strategy**

A number of researchers argue that changing an established lifestyle in the short term is difficult. One study reported the traditional approach to reduce energy intake to improve eating behavior and increase energy output from physical activity in company and community based health classes induced a high rate of rebound to the former body weight. However, excessive calorie intake or physical inactivity is extremely small. Previous nutritional research in the U.S. showed that a reduction of only 100 kcal per day from improved energy intake–expenditure would contribute to an inhibition of 90% of the population’s weight gain, even though obesity is a rising social problem in the American population. The advantages of applying the small change strategy to improve physical activity and healthy eating are that they (a) are realistic and sustainable, (b) aid in weight control even though the lifestyle change is small, (c) contribute to an increase in self-confidence or efficacy after one succeeds in small lifestyle changes.

**Examples of the small change strategy to improve physical activity and healthy eating habits**

Below table shows the example of the small change of being able to practice during daily living.

**Table** Examples of small lifestyle changes

| Physical activity | Eating behavior |
| --- | --- |
| Turn a coffee break into a stretch break | Eat healthier boiled rather than grilled meat |
| Walk in long strides | Eat fruit instead of sweets |
| Go shopping at a store that is a little further away | Reduce chocolate to two pieces from four pieces |
| Walk for 5 minutes longer than usual | Eat moderately and never fill your stomach |
| Mop the floor and generally clean more frequently than at present | Reduce drinking at dinner from every day to three times a week |
| Divide the trash and put it out more often | Eat deep-fried food only once a week |
