# Supplementary material for: Emotional experiences of reading health educational manga encouraging behavioral changes: a non-randomized controlled trial
Source: Health Psychol Behav Med. 2021 Apr 30;9(1):398–421. doi: 10.1080/21642850.2021.1921583 (PMC8159205; doi:10.1080/21642850.2021.1921583)
Supplement: Supplemental Material [file RHPB_A_1921583_SM1419.zip › Additional file 3.docx]

**Additional file 3. Assessed items and constructive concept (English translation)**

**1. Emotional experience according to read health educational manga (Hypothesized item)**

Instruction: This time questions about the content of health information will be asked. Please encircle (◯) only one letter of the alphabet that is the most applicable for each of the following questions:

(1: Do not agree at all,　2: Do not agree much, 3: Neither, 4: Agree, and 5: Absolutely agree)

| Hypothesized concept | Item |
| --- | --- |
| Enjoy | I was able to enjoy reading the contents of the health information. |
| Fun | I felt that the content of the health information was interesting. |
| Prize | I was happy to know about health. |
| Satisfaction | I was satisfied to know about health. |
| Crisis | I felt a sense of crisis with respect to my health condition. |
| Intemperance | I realized my neglect of health. |
| Anxiety | I felt uneasy about my future health condition. |
| Self-monitoring | It provided the inspiration to think about the current state of my own health situation. |
| Foresight | I imagined my future in which I continued my current lifestyle. |
| Pros | I thought about the benefits of implementing the recommended actions. |
| Cost | I thought about the loss sustained due to not implementing the recommended actions. |
| Social Norm | I thought about the effects of engaging in unhealthy behavior on family and friends. |
| Story familiarity | I felt the content of the story was familiar. |
| Empathy | I empathized with the speech and behavior of the characters. |
| Identification | The speech and behavior of the characters felt as if they were my own. |
| Transportation | I empathized with the characters. |
| Story realism | I felt that the content was realistic. |
| Character realism | I felt that the characters were likely to exist in reality. |
| Realistic | I felt that the recommended content was realistic. |
| Relativeness | I felt that the content is also related to my current self. |

**2. Acceptability-Usability scale for assessing health information media**

**1）Instruction**

This time, questions about the content of health information will be asked. Please circle (◯) only the letter that is most applicable for each of the following questions:

| Superordinate | Subordinate | Item |
| --- | --- | --- |
| Acceptability | Comprehensiveness | 1. How did you feel about the comprehensiveness of the present brochure?  (1: Did not understand at all; 2: Did not understand much; 3: Unsure; 4: Understandable; and 5: Very understandable) |
|  | Familiarity | 2. Were the phrasing, illustration, and charts in the provided information familiar to you?  (1: Not familiar at all; 2: Not very familiar; 3: Unsure; 4: Familiar; and 5: Very familiar) |
|  | Acceptance of media | 3. Was the present information type (using the Internet) acceptable to you?  (1: Not acceptable at all; 2: Not very acceptable, 3: Unsure, 4: Acceptable, and 5: Very acceptable) |
| Usability | Understanding benefit | 4. Did you understand the benefits that you would obtain by engaging in healthy behavior?  (1: Did not understand at all; 2: Did not understand much; 3: Unsure; 4: Understandable; and 5: Very understandable) |
|  | Perceived content efficiency | 5. How useful was the information material for you?  (1: Not useful at all; 2: Not very useful; 3: Unsure; 4: Useful; and 5: Very useful) |
|  | Personal relevance | 6. How closely related was the content of the information to your own understanding of what you thought you should do?  (1: Not suitable at all; 2: Not very suitable; 3: Unsure; 4: Suitable; and 5: Very suitable) |

Reference:

Shimazaki, T., Maeba, K., Iio, M., Takenaka, K., & Kikkawa, M. (2013). Acceptability and usability of health communications on changing health behaviors and self-efficacy. *Japanese Journal of Health Psychology*, *26*(1), 7–17 (in Japanese).

**2）Reliability and validity of measured scale on present study participants (n = 1,640)**


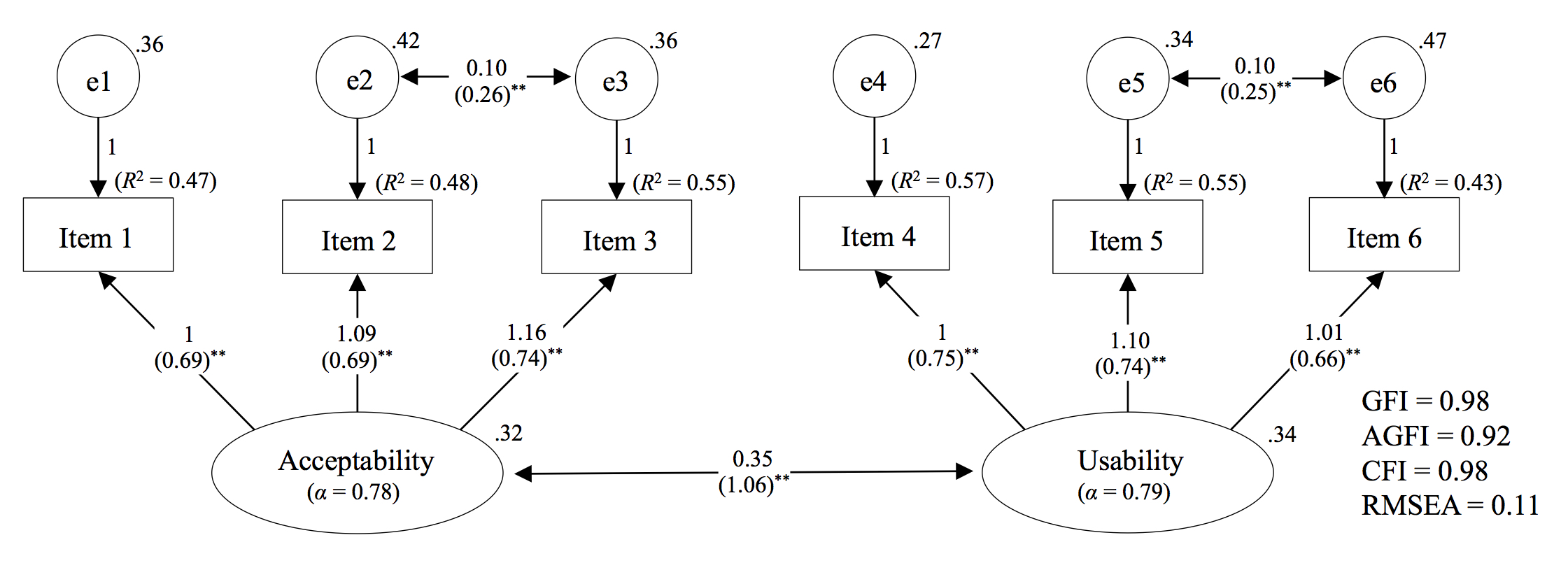


Fig. A3-1 Acceptability-usability scale for assessing health information media

**3. Primary outcomes: Self-efficacy and behavioral intention scale**

**1）Instruction**

This time questions about changes in your own self, regarding reading of the health information presented will be asked. Please encircle only one letter of the alphabet that is the most applicable for each of the following questions.

(1: Do not agree at all,　2: Do not agree much, 3: Neither, 4: Agree, and 5: Absolutely agree)

| Superordinate | Subordinate | Item |
| --- | --- | --- |
| Self-efficacy | Confidence | 1. I was confident of continuing my healthy behavior. |
|  | Simplicity | 2. I thought it was easy to enforce healthy behavior. |
|  | Controllability | 3. I thought it was up to me whether I could enforce healthy behavior or not. |
| Behavioral intention | Expectation | 4. I thought I will try and enforce healthy behavior from now, or from now also. |
|  | Desire | 5. I thought of enforcing healthy behavior |
|  | Planning | 6. I thought of making a plan to enforce healthy behavior. |

Reference:

Francis J, Eccles MP, Johnston M, Walker AE, Grimshaw JM, Foy R, Kaner EFS, et al. Constructing questionnaires based on the theory of planned behaviour: A manual for health services researchers. Centre for Health Services Research. 2004. http://openaccess.city.ac.uk/1735/

**2）Reliability and validity of measured scale on present study participants (n = 1,640)**


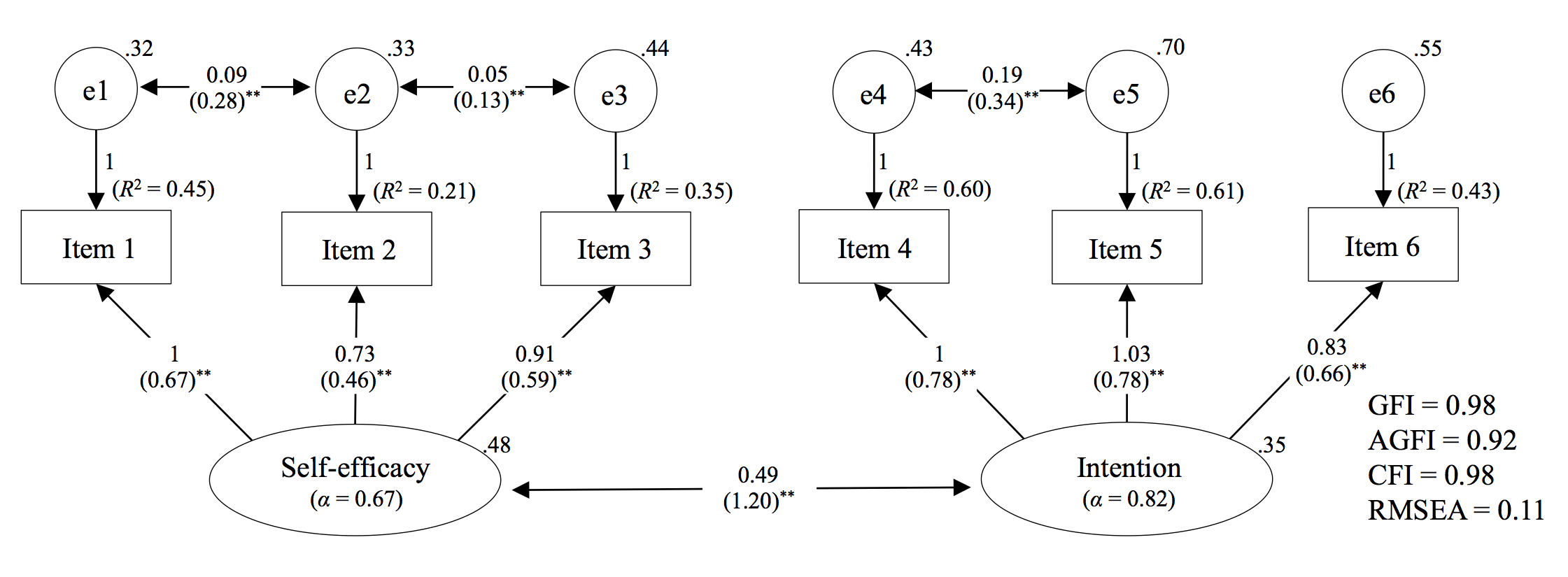


Fig.A3-2 Self-efficacy and behavioral intention scale

**4. Secondary outcomes: Antecedent factor of health behavior**

This time questions about changes in your own self, regarding reading of the health information presented will be asked. Please encircle ◯ only one letter of the alphabet that is the most applicable for each of the following questions.

(1: Do not agree at all,　2: Do not agree much, 3: Neither, 4: Agree, and 5: Absolutely agree)

| Superordinate | Subordinate | Item |
| --- | --- | --- |
| Motivation |  | 1. It led to the willingness (motivation) to enforce healthy behavior. |
| Attitude/ Belief |  | 2. There was a good influence on my way of thinking and my attitude towards health. |
| Subjective norm |  | 3. I could understand how others around me are thinking about unhealthy behavior. |
| Knowledge |  | 4. Knowledge about health promotion increased |
| Recall |  | 5. I remembered methods of promoting health. |
