# Supplementary material for: Emotional experiences of reading health educational manga encouraging behavioral changes: a non-randomized controlled trial
Source: Health Psychol Behav Med. 2021 Apr 30;9(1):398–421. doi: 10.1080/21642850.2021.1921583 (PMC8159205; doi:10.1080/21642850.2021.1921583)
Supplement: Supplemental Material [file RHPB_A_1921583_SM1419.zip › Additional file 4.docx]

**Additional file 4. Relationship between the independent factors of emotional experience when reading educational health manga and readiness for behavior change**


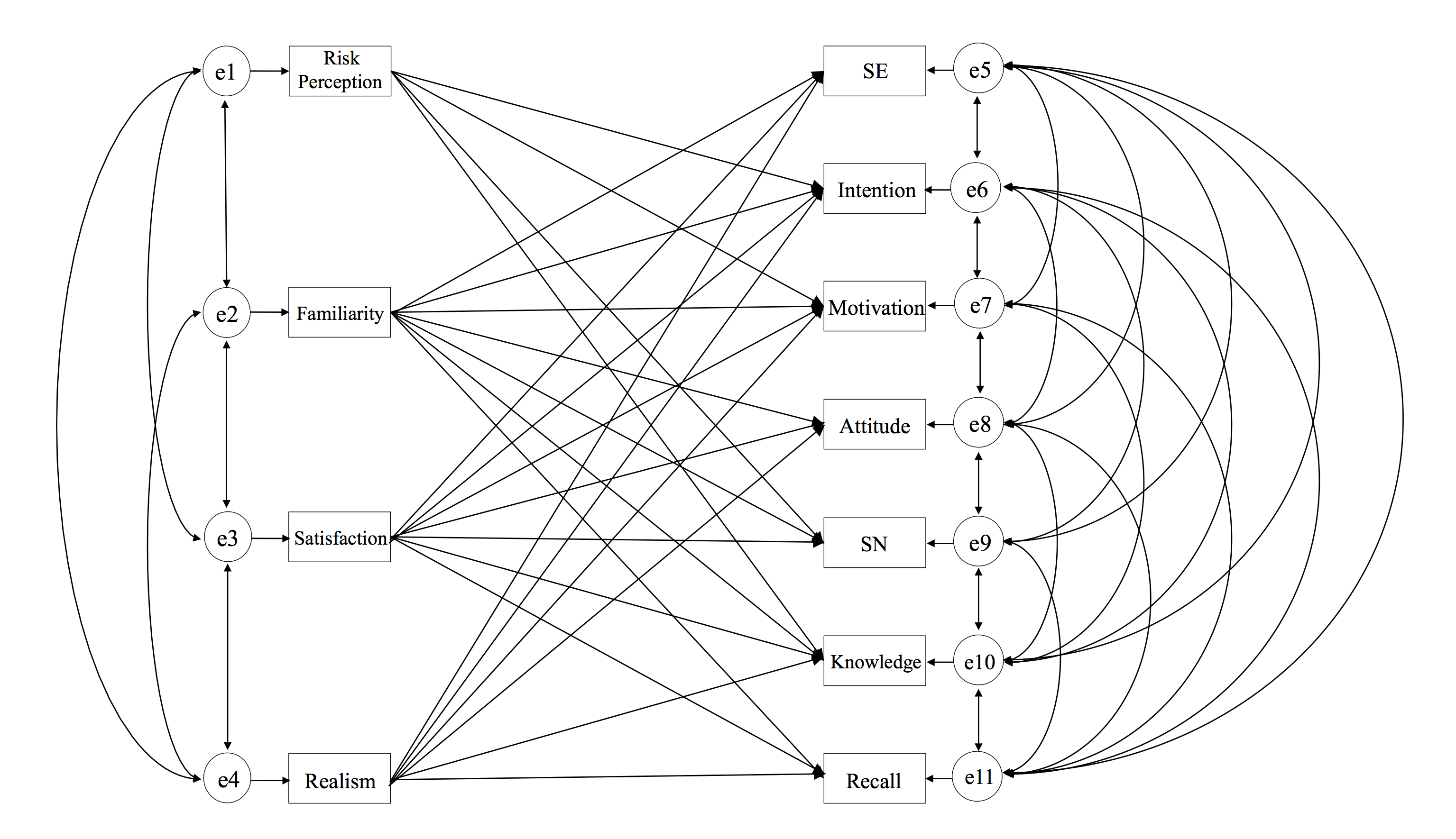


*Figure A4*: Relationship between the independent factors of emotional experience and readiness for behavior change

*Note*. Results of the structural equation model are shown in Tables A3.1 and A3.2. Only significant paths were demonstrated. SE: self-efficacy, SN: subjective norm.

*Table A4.1*: Path coefficient of the accepted model for a relationship between the four factors of emotional experience and psychological readiness

|  |  | Dependent variables | | | | | | |
| --- | --- | --- | --- | --- | --- | --- | --- | --- |
|  | Path coefficient | Motivation | Attitude | SN | SE | Intention | Knowledge | Recall |
| Independent variables | |  |  |  |  |  |  |  |
| Risk perception | Unstandardized | 0.17 | *n.s* | 0.25 | *n.s* | 0.16 | 0.09 | *n.s* |
|  | (Standardized) | (0.14)^**^ |  | (0.24)^**^ |  | (0.17)^**^ | (0.08)^*^ |  |
| Familiarity | Unstandardized | 0.23 | 0.21 | 0.20 | 0.26 | 0.21 | 0.21 | 0.26 |
|  | (Standardized) | (0.20)^**^ | (0.20)^**^ | (0.18)^**^ | (0.28)^**^ | (0.21)^**^ | (0.18)^**^ | (0.24)^**^ |
| Satisfaction | Unstandardized | 0.44 | 0.47 | 0.25 | 0.29 | 0.38 | 0.49 | 0.38 |
|  | (Standardized) | (0.36)^**^ | (0.43)^**^ | (0.22)^**^ | (0.31)^**^ | (0.38)^**^ | (0.42)^**^ | (0.34)^**^ |
| Realism | Unstandardized | 0.11 | 0.18 | *n.s* | 0.14 | 0.14 | 0.10 | 0.26 |
|  | (Standardized) | (0.09)^*^ | (0.17)^**^ |  | (0.16)^**^ | (0.14)^**^ | (0.09)^*^ | (0.24)^**^ |
|  | *R*^2^ | 0.50 | 0.52 | 0.32 | 0.47 | 0.63 | 0.48 | 0.55 |

*Note.* SE: Self-efficacy; SN: Subjective norm

^*^*p* < 0.05, ^**^*p* < 0.01

*Table A4.2*: Covariance in error variables of the accepted model for a relationship between the four factors of emotional experience and psychological readiness

|  |  | e1 | e2 | e3 | e4 | e5 | e6 | e7 | e8 | e9 | e10 | variance |
| --- | --- | --- | --- | --- | --- | --- | --- | --- | --- | --- | --- | --- |
| e1 | covariance |  |  |  |  |  |  |  |  |  |  | 0.62 |
|  | ( *r* ) |  |  |  |  |  |  |  |  |  |  |  |
| e2 | covariance | 0.35 |  |  |  |  |  |  |  |  |  | 0.59 |
|  | ( *r* ) | (0.59)^**^ |  |  |  |  |  |  |  |  |  |  |
| e3 | covariance | 0.40 | 0.42 |  |  |  |  |  |  |  |  | 0.57 |
|  | ( *r* ) | (0.67)^**^ | (0.73)^**^ |  |  |  |  |  |  |  |  |  |
| e4 | covariance | 0.40 | 0.44 | 0.42 |  |  |  |  |  |  |  | 0.60 |
|  | ( *r* ) | (0.66)^**^ | (0.74)^**^ | (0.73)^**^ |  |  |  |  |  |  |  |  |
| e5 | covariance |  |  |  |  |  |  |  |  |  |  | 0.25 |
|  | ( *r* ) |  |  |  |  |  |  |  |  |  |  |  |
| e6 | covariance |  |  |  |  | 0.12 |  |  |  |  |  | 0.22 |
|  | ( *r* ) |  |  |  |  | (0.50)^**^ |  |  |  |  |  |  |
| e7 | covariance |  |  |  |  | 0.08 | 0.12 |  |  |  |  | 0.41 |
|  | ( *r* ) |  |  |  |  | (0.25)^**^ | (0.39)^**^ |  |  |  |  |  |
| e8 | covariance |  |  |  |  | 0.10 | 0.11 | 0.09 |  |  |  | 0.33 |
|  | ( *r* ) |  |  |  |  | (0.35)^**^ | (0.41)^**^ | (0.26)^**^ |  |  |  |  |
| e9 | covariance |  |  |  |  | 0.10 | 0.09 | 0.03 | 0.09 |  |  | 0.48 |
|  | ( *r* ) |  |  |  |  | (0.30)^**^ | (0.29)^**^ | (0.07)^*^ | (0.23)^**^ |  |  |  |
| e10 | covariance |  |  |  |  | 0.09 | 0.10 | 0.11 | 0.09 | 0.09 |  | 0.41 |
|  | ( *r* ) |  |  |  |  | (0.28)^**^ | (0.34)^**^ | (0.26)^**^ | (0.25)^**^ | (0.20)^**^ |  |  |
| e11 | covariance |  |  |  |  | 0.11 | 0.09 | 0.06 | 0.10 | 0.07 | 0.10 | 0.33 |
|  | ( *r* ) |  |  |  |  | (0.30)^**^ | (0.34)^**^ | (0.18)^**^ | (0.30)^**^ | (0.17)^**^ | (0.27)^**^ |  |

^**^*p* < 0.01
